# The boon and bane of boldness: movement syndrome as saviour and sink for population genetic diversity

**DOI:** 10.1186/s40462-020-00204-y

**Published:** 2020-04-21

**Authors:** Joseph Premier, Jörns Fickel, Marco Heurich, Stephanie Kramer-Schadt

**Affiliations:** 1 Chair of wildlife ecology and wildlife management, Faculty of Environment and Natural Resources, University of Freiburg, Tennenbacher Straße 4, 79106 Freiburg, Germany; 2 Leibniz Institute for Zoo and Wildlife Research (IZW), Alfred-Kowalke-Str. 17, 10315 Berlin, Germany; 3 Department of Visitor Management and National Park Monitoring, Bavarian Forest National Park, Freyunger Str. 2, 94481 Grafenau, Germany; 4 Institute for Biochemistry and Biology, University of Potsdam, Karl-Liebknecht-Str. 24-25, 14476 Potsdam-Golm, Germany; 5 Department of Ecology, Technical University Berlin, Rothenburg Str. 12, 12165 Berlin, Germany

## Abstract

**Background:**

Many felid species are of high conservation concern, and with increasing human disturbance the situation is worsening. Small isolated populations are at risk of genetic impoverishment decreasing within-species biodiversity. Movement is known to be a key behavioural trait that shapes both demographic and genetic dynamics and affects population survival. However, we have limited knowledge on how different manifestations of movement behaviour translate to population processes. In this study, we aimed to 1) understand the potential effects of movement behaviour on the genetic diversity of small felid populations in heterogeneous landscapes, while 2) presenting a simulation tool that can help inform conservation practitioners following, or considering, population management actions targeting the risk of genetic impoverishment.

**Methods:**

We developed a spatially explicit individual-based population model including neutral genetic markers for felids and applied this to the example of Eurasian lynx. Using a neutral landscape approach, we simulated reintroductions into a three-patch system, comprising two breeding patches separated by a larger patch of differing landscape heterogeneity, and tested for the effects of various behavioural movement syndromes and founder population sizes. We explored a range of movement syndromes by simulating populations with various movement model parametrisations that range from ‘shy’ to ‘bold’ movement behaviour.

**Results:**

We find that movement syndromes can lead to a higher loss of genetic diversity and an increase in between population genetic structure for both “bold” and “shy” movement behaviours, depending on landscape conditions, with larger decreases in genetic diversity and larger increases in genetic differentiation associated with bold movement syndromes, where the first colonisers quickly reproduce and subsequently dominate the gene pool. In addition, we underline the fact that a larger founder population can offset the genetic losses associated with subpopulation isolation and gene pool dominance.

**Conclusions:**

We identified a movement syndrome trade-off for population genetic variation, whereby bold-explorers could be saviours - by connecting populations and promoting panmixia, or sinks - by increasing genetic losses via a ‘founder takes all’ effect, whereas shy-stayers maintain a more gradual genetic drift due to their more cautious behaviour. Simulations should incorporate movement behaviour to provide better projections of long-term population viability and within-species biodiversity, which includes genetic diversity. Simulations incorporating demographics and genetics have great potential for informing conservation management actions, such as population reintroductions or reinforcements. Here, we present such a simulation tool for solitary felids.

## Background

Small populations of endangered species are of high conservation concern. Not only are they at risk due to demographic stochasticity, which can lead to extinction [1, 2], but isolation also puts them at risk of genetic drift and inbreeding [3]. These processes erode species’ genetic diversity and thus their intrinsically encoded phenotypic diversity [4], which are key components of biodiversity (Convention on Biological Diversity, Article 2) [5]. In human-dominated ecosystems, environmental changes have accelerated, and organisms must adapt to these changes to persist. Functional response traits are the expression of a two-way interplay between evolutionary adaptation to environmental conditions and the plasticity of species or individuals [6]. Restricting the responses, in other words phenotypes, can negatively impact species’ adaptability [7]. In this context, movement is a key functional trait that has a decisive role, not only in determining an individual’s fate, but also in mitigating population isolation. Certain movement traits, such as distance or propensity, could help connect populations and protect within-species diversity, which makes movement of great importance when predicting long-term population viability.

Movement rates, i.e. the outcome of individual movements, are known to drive both genetic differentiation and homogenisation in subdivided populations (e.g. [8]). As effective movement rates depend on the permeability of the environment, the spatial structure of landscape is another important factor shaping the genetic differentiation between and within populations (e.g. [9]). Thus, genotypes subsume the effects of movement in heterogeneous landscapes, population establishment and genetic exchange and, therefore, genetic metrics such as F-statistics or heterozygosity can reveal detailed information on population viability.

The individual expression of behavioural responses, named variously “personality” [10] or “behavioural syndrome/ type” [11], is of great importance for adaptability to environmental changes. Behavioural syndromes are characterised by consistent and repeatable within-species differences on various response axes, including aggression, boldness, and sociability [12]. Variability of movement traits in this context has been shown both experimentally and empirically (e.g. [13, 14]). However, it is still unclear to what extent these behavioural movement syndromes (hereafter movement syndromes) can influence the development of population genetic diversity and structure in heterogeneous landscapes. This knowledge would help broaden the characterisation of population viability beyond demography. Hence to explore these processes, genetic markers should be combined with methods that deal explicitly with landscape heterogeneity and movement complexity at the individual level.

Spatially explicit individual-based models (SEIBMs) are useful tools for exploring complex systems for which dynamics at the population level emerge from individual decision-making [15]. Recently, individual-based processes have been included in spatially explicit demographic-genetic, or “demogenetic”, population simulations (e.g. fish [16], trees [17], birds [18], and rodents [19]). Demogenetic models include the assignment of neutral genetic markers to individuals’ properties. Their empirical counterparts, e.g. microsatellite loci, are used to monitor genetic drift and inbreeding and, although neutral genetic variation is not associated with any given phenotypic variability or functional traits, they are well suited for studying genetic diversity and differentiation [20]. Neutral genetic variation can also be assumed to reflect an individual’s fitness [21]. Therefore, these models are appropriate for analyses of demogenetic responses [22]. Using a neutral landscape approach [23] with a demogenetic model featuring mechanistic movement, it is possible to disentangle the effects of movement syndromes and landscape heterogeneity on the population genetics of a study species (e.g. [18]). However, as argued by Bruggeman et al. [18], species specific models must be used to correctly address scaling issues of movement decisions and life histories in concert with landscape heterogeneity.

We chose a felid as our study species due to the conservation concern of several species in this family, e.g. Iberian lynx (*Lynx pardinus*) [24], Texas ocelot (*Leopardus pardalis albescens*) [25] or tiger (*Panthera tigris*) [26], especially regarding genetics and often caused by landscape factors. In addition, since most felids have similar land tenure systems, social and life-history traits [27, 28] inferences are transferable, however, with caveats e.g. for scale. We selected Eurasian lynx (*Lynx lynx*) (hereafter lynx) because of its high relevance in the wake of many population reintroductions in Central Europe [29–33]. Despite demographically viable reintroduced sub-populations, small population sizes in combination with isolation currently puts lynx populations at risk due to genetic drift, i.e. random loss of alleles and possible fixing of deleterious alleles, and potential inbreeding depression [3, 34]. These factors can negatively affect individual fitness, which ultimately affects population viability [35]. Therefore, maintaining genetic diversity is crucial for retaining lynx’s adaptive potential. According to the personality-dependent spatial ecology framework [36] population level patterns emerge due to movement syndromes and their interactions with landscape. However, until now their combined effects on the population genetics of reintroduced lynx populations had not been studied and were therefore unknown. Furthermore, a combined demographic and genetic (i.e., demogenetic) model appropriate for studying felids in a spatial context, i.e. under habitat fragmentation and including movement, was missing.

In this study, we present a demogenetic model for felids based on an existing SEIBM [37], with the goals: 1) to understand the potential effects of landscape heterogeneity, movement syndromes and founder population size, on the genetic diversity and structure of lynx populations, and 2) to help conservation managers considering, or following, population reintroductions and reinforcements of felids by offering a suitable population simulation tool. We achieve these goals by applying the model developed I) in a neutral landscape framework and II) under control scenarios with no emigration for comparison, to confront our research question (1), and as a proof of concept for the model’s application (2).

## Methods

An existing individual-based, spatially explicit felid population model [37] was expanded and used to simulate the spatiotemporal dynamics of lynx population genetics in neutral landscapes. The existing model consists of several submodules covering dispersal, demographics and habitat preference [37, 38], all of which were previously calibrated for lynx with field data [39]. In this study we used theoretical landscape maps analysed previously [40], which were derived using a neutral landscape model. Finally, a genetic module has been implemented to simulate neutral genetic markers. All the model’s spatial operations share the resolution of the landscape grid (total = 149 × 65 cells), where each cell represents 1km^2^.

The demogenetic model simulates felid life history stages following the “felid year” cycle (start/end coincides with independence of sub adults and the onset of breeding season). At initialisation and at the start of each time step (felid year) a population census records the demogenetic status of all individuals. Thereafter, all non-resident animals older than 1 year (age of independence) disperse in search of unoccupied territories. Dispersal (see dispersal submodule) and territory selection (see ODD supplementary material S1) are spatially explicit processes that depend upon individual experience of local habitat quality. These processes are regulated by rules which consider the habitat in cells adjacent to an individual’s location. If dispersing individuals survive, they either settle, given suitable territories, or they continue dispersal in the next year. Next, we determine for each resident female whether there is reproduction (see population submodule) and, given reproduction, inheritance and mutation of neutral genetic markers is handled (see genetic submodule). Finally, we update the demographic variables for each surviving individual (age and status, i.e., disperser or resident) before the next simulation time step begins.

An overview of the novel and most relevant submodules is given in the following sections and a detailed description of these and other submodules is found in the ODD protocol (supplementary material S1). For values of parameters and variables of the model mentioned in the submodules below see Table 1.

**Table 1:** Input parameter sets and outputs of demogenetic simulations and variable importance (%incMSE) and rank (from highest to lowest variable importance) for 5 separate RF metamodels with input parameters as independent variables and outputs as dependent variables. Simulations carried out in a three-patch landscape with “source” and “arrival” breeding habitat patches separated by a “connectivity” patch with varying amounts of ‘dispersal habitat’ and varying ‘degrees of fragmentation’ (i.e. from 1 for randomly distributed dispersal habitat to 4 for large blocks of continuous dispersal habitat), for 3 different movement syndromes (MS 1: shy, MS 2: intermediate, MS 3: bold) and 3 sizes of the founder population

Demogenetic model outputs/RF metamodel dependent variables (observations)	Demogenetic model parameters/RF metamodel independent variables							
	Number of Founders: 10, 50, 100 [initial population size]		Movement syndrome: Shy (MS.1), intermediate (MS.2), bold (MS.3) [shy-bold continuum] respectively; P_matrix_ =0.03, 0.06, 0.12; P_c_ =50, 50, 50; P_maxmat_ =10, 20, 40		Amount of dispersal habitat: 0 (CS1), 10, 20, 30, 40, 60, 80, 100 (CS2) [%]		Degree of fragmentation: 1, 2, 3, 4 [random - clumped continuum]	
	%incMSE	Rank	%incMSE	Rank	%incMSE	Rank	%incMSE	Rank
**H**_**o**_ (163,862)	473.16	**1**	253.45	4	346.39	2	343.37	3
**F**_**ST**_ (104,082)	13.04	4	238.64	2	312.30	**1**	205.98	3
**F**_**IS**_ (104,082)	8.08	4	51.61	2	81.97	**1**	49.59	3
**Source patch λ** (163,862)	0.91	2	−0.08	3	318.99	**1**	−1.04	4
**Arrival patch λ** (84,967)	300.92	**1**	88.98	3	170.52	2	83.46	4
**Arrival patch Colonisation** (163,862)	−0.14	4	170.38	3	265.18	**1**	183.13	2

### Dispersal submodule

In this work we employed an individual-based spatially explicit dispersal model (SEDM) based on a set of intraday behavioural rules [41]. The SEDM is applied on landscapes defined by 1 km^2^ grid cells, with each grid cell attributed one of four habitat types: breeding habitat, dispersal habitat, unsuitable (avoided) habitat (matrix), or barrier (uninhabitable). The emerging dispersal movement patterns are functions of the spatial distribution of habitat types and the probability distributions that define individuals’ movement decisions.

Individuals may move 1 grid cell per step, with a total number of per day steps, *s*, taken anew each day from the probability distribution P(*s*) defined by the power function:

$$ \mathrm{P}(s)=\phi {\left(1-\left(\frac{s-1}{s_{\mathrm{max}}-1}\right)\right)}^x $$

with exponent *x* which controls the function’s steepness and rarity of long-distance movements, *s*_max_ the maximum number of intraday steps for dispersing individuals and *ϕ* a normalization factor which scales the probabilities between 0 and 1. The probability density function P(*s*) describes the probability of taking a certain number of intraday steps and is based on the empirical distribution of daily movement distances obtained from rigorous inverse model fitting [39, 41].

A correlated habitat-dependent walk is used to model the movement of individuals under the assumption that they perceive the habitat quality in the adjacent cells. The probability of stepping into any one of the adjacent grid cells is determined based on the preference for ‘dispersal habitat’. Dispersing individuals have equal preference for breeding and dispersal habitats. If only dispersal or matrix habitat is available (where availability is defined as the 8 adjacent grid cells plus individual’s location) the next cell is selected randomly (i.e. 1/9). The probability of selecting a given available ‘matrix habitat’ cell when ‘dispersal habitat’ is also available is P_matrix_. This variable allows lynx to step into matrix from time to time, even when dispersal habitat is available. A correlation factor, P_c_, was applied giving the probability that movement direction is maintained within 1 day, for which P_c_ = 0 implies random direction and P_c_ = 1 direction maintained. The direction of the first step each day is randomly assigned. ‘Dispersal habitat’ is preferred before correlation of movement direction is considered. Finally, the maximum number of consecutive grid cells an individual can travel in matrix habitat is defined by P_maxmat_. This limits unrealistically long movement distances across matrix by assuming that forays into avoided habitat will be aborted at some distance, at which point the individual will return to the last dispersal habitat grid cell it occupied.

### Population submodule

The demographic model [37] contains processes that depend on the individual’s status, as either resident or disperser. The model also considers differences in gender and individual variation when determining territory selection and extent (i.e. delineation of an individual’s territory within the landscape). In brief, territory selection of dispersing females depends on finding an unoccupied area of breeding habitat (minimum 46 km^2^, maximum 146 km^2^). Dispersing males must find territories of resident females where no resident male is present. Male territories can overlap with up to three female territories (see ODD in supplementary materials S1), making male-biased dispersal distances an emerging property of the model. Mortality is evaluated daily for dispersers and yearly for residents with probabilities, P_MD_ 0.0015/day (≡ 0.547/year) and P_MR_= 0.1/year respectively, taken from a previous empirical study [37]. According to Kramer-Schadt et al. [37], there is no evidence that mortality rates of dispersers were higher in matrix than dispersal habitat, but mortality of dispersers was higher due to roads. We subsumed the road mortality into P_MD_, the daily mortality rate of dispersers, to reflect this and to exclude a confounding relation between mortality and connectivity. Residents may produce offspring with a given probability (P_B_= 0.75) when female (older than 2 years) and male territories overlap. Litter size and kitten mortality probabilities were defined to give on average 1.5 independent sub adults per reproduction event at the start of the next year. Kittens inherit the genetics of their parents (see below ‘genetic submodule’).

### Genetic submodule

**Fig. 1:**
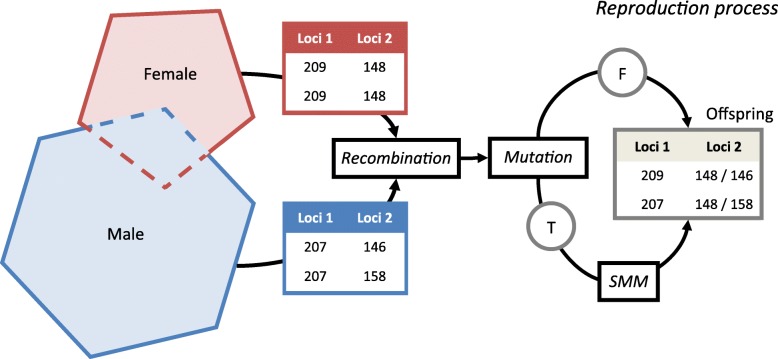
Schematic representation of reproduction process for adult males and females with overlapping territories, showing an offspring’s potential allele inheritance at two exemplary loci without mutation (F). Given mutation (T) this is handled using the ‘stepwise mutation model’ (SMM)

A genetic module for the distribution of neutral genetic markers was implemented to allow Mendelian inheritance and genetic mutation in the reproduction procedure of the population model (Fig. 1). Neutral genetic markers are gene loci not associated with fitness-related functional traits or subject to selection processes and are hence suitable for inferences between genetic structure and landscape, as shown in a wide range of studies (e.g. [42]). Genetic properties of individuals were implemented as diploid genotypes based on 12 loci (i.e. 12 allele pairs). This number has been used in empirical population genetic studies on lynx [34] and is sufficient for determining spatial genetic patterns [43]. Alleles are stored as integer numbers which represent microsatellite lengths. During reproduction, offspring inherit one allele per parent per loci, with an even probability given to each parental allele. This gives a total of 24 alleles - 12 paternal and 12 maternal alleles.

Genetic mutation is simulated using a stepwise mutation model (SMM) [44]. During reproduction the inherited alleles undergo mutation at a given rate, set at 10^− 4^ per locus per generation [45]. If mutation of an allele takes place, the allele’s integer value A_0_ has an even probability of increasing or decreasing its value by 1, to A_0_+ 1 or A_0_− 1. The lower limit is constrained as a microsatellite length of zero is impossible, hence if A_0_= 1 a length decreasing step is not permitted and an even probability is assumed for either increasing or keeping the allele length.

### Simulation experiments

**Fig. 2:**
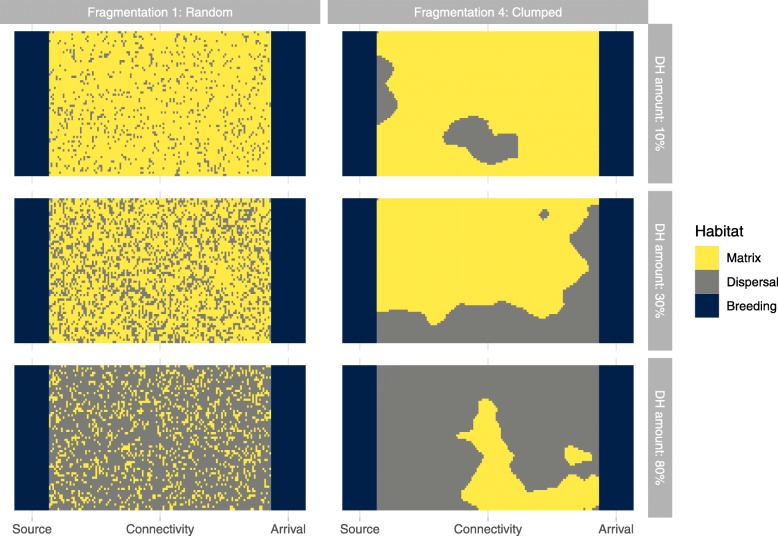
Neutral landscapes with “source” and “arrival” breeding patches separated by a varying “connectivity” patch with differing configurations of ‘matrix habitat’ and ‘dispersal habitat’, exemplified with ‘dispersal habitat’ amounts 10–80% coverage and ‘degree of fragmentation’ values 1 (randomly distributed dispersal habitat) and value 4 (large blocks of continuous dispersal habitat). Twenty-five iterations of each landscape were used

We simulated lynx reintroductions in a three-patch system, with two patches of breeding habitat separated by a larger “connectivity” patch of mixed dispersal and matrix habitat types (Fig. 2). Founder animals were released in the so-called “source” breeding patch, with the “arrival” breeding patch initially unoccupied. Three movement “syndromes” (MS) were studied independently along a ‘shy stayer’ and ‘bold mover’ continuum. In this study, the movement of MS 1 was defined by a SEDM parameter set calibrated previously, via analysis of an empirical telemetry dataset [41]. MS 2 and MS 3 had values designed to increase their boldness; i.e. higher P_maxmat_ and P_matrix_ (Table 1). This leads to a higher preponderance for, and longer, excursions in ‘matrix habitat’. Therefore, the MS are referred to as shy (MS1), intermediate (MS2) and bold (MS3) after their relative positions along the shy-bold continuum.

We used artificially created neutral landscapes to analyse the effect of landscape characteristics on genetic diversity. Neutral landscapes are computer generated models of landscape patterns that are commonly used to answer questions in landscape ecology [46]. The landscapes (examples in Fig. 2) comprised two breeding habitat patches, “source” and “arrival”, (each 20 × 65 cells, i.e. 1300 km^2^) that were constant in all landscape scenarios. Separating them was a larger “connectivity” patch (129 × 65 cells, i.e. ca. 11,000 km^2^), within which the proportion and configuration of dispersal and matrix habitats was varied. This landscape setup represents realistic situations, such as the size and separation of some low mountain ranges suitable for hosting lynx populations in Central Europe (e.g. separation of Thuringian and Bavarian-Bohemian Forests), and has been used previously [40]. The breeding patch size leads to an emergent value of approximately 30 resident individuals per patch. Six amounts of ‘dispersal habitat’ (= 10, 20, 30, 40, 60, and 80%) and four degrees of habitat fragmentation (ranging from 1 - randomly distributed to 4 - clumped) were used. The configuration of ‘dispersal habitat’ was created using the midpoint displacement algorithm [47], with the remaining cells filled in with ‘matrix habitat’ All possible combinations of fragmentation and ‘dispersal habitat’ amount were produced (4 × 6 = 24 landscapes), with 25 replications to allow for the stochastic spatial arrangement of the landscape algorithm. The population simulations were repeated 30 times per landscape (i.e. 25 × 30 = 750 repetitions per landscape parameter set). Three additional landscapes were included as control scenarios (CS), for which the “connectivity” patch was filled with: CS1–100% ‘matrix habitat’, CS2–100% ‘dispersal habitat’, and CS3–100% ‘barrier’. Barrier refers to a habitat type which individuals cannot move into or through, it appears only in CS3. CS1 and CS2 describe all values of fragmentation and constrain the limits of worst and best ‘dispersal habitat’ respectively. CS3 restricts movements to the “source” patch and thus describes population genetics in a closed patch. For further details pertaining to the neutral landscapes, see Kramer-Schadt et al. [40].

The founder population (size = 10, 50 or 100 individuals) was introduced into the “source” patch, with individual starting locations randomly distributed in the patch. Gender distribution was even in all cases. The genotypes of the founders were intended to represent a “near perfect” reintroduction, i.e. where individuals are randomly chosen from a large autochthonous population, and hence are assumed to be unrelated. This leads uniformly to high initial individual and population genetic diversity across all simulation runs (emergent founder population H_o_≈ 1 in first year), thereby circumventing additional complexity associated with relatedness in real founding events. To simulate allele lengths, we assigned all individuals 24 alleles with integer values uniformly sampled (with replacement) between 120 and 220, based on empirical data ranges [34]. A set of 30 founder populations (i.e. 1 per repetition of each simulation parameter set, see above), per founder population size (total 90 populations), were produced to give stochastic genetic structure at initialisation of each simulation repetition.

The parameter sets for each movement syndrome (3 levels; Table 1) and founder population size (3 levels; 10, 50 and 100 individuals) were simulated on all landscapes (i.e. 3 movement syndromes × 3 initial population sizes [[24 landscape configurations repeated 25 times] + 3 control scenarios] × 30 simulation repetitions = 162,810 simulation runs), with the population simulations lasting 200 years (approx. 40 generations). The landscape boundaries were reflective, to prevent emigration from the system, as could be expected in regions where barrier features such as industrial/urban areas border more natural areas. In year 200 all individuals were recorded for computation of the model outputs.

### Model outputs

As we were interested in the effect of landscape type and movement syndrome on genetic diversity, the metrics used to determine the gene (allele) pool were calculated at the total population level, i.e. individuals from both source and arrival patches were included. Only living resident individuals were considered in the population genetics, as only residents took part in reproduction. A maximum of 30 residents per patch were selected for genetic analyses (randomly if residents per patch> 30). All analyses were conducted in the statistical software R [48].

The genetic metrics, ‘observed heterozygosity’ (H_o_), used as a proxy for genetic diversity, and F-statistics (population genetic differentiation and inbreeding coefficient, respectively F_ST_ and F_IS_), were computed with functions from the R package “adegenet” [49]. A diploid (2 allele) locus is homozygous (H_o_ = 0) if the alleles are identical, and heterozygous (H_o_ = 1) if the alleles differ. When calculated for a population, higher H_o_ (→1) indicates greater heterogeneity of loci and higher diversity, and vice versa for lower H_o_ (→0). F_ST_, the fixation index, is calculated as the proportion of allelic variance that can be explained by population structure, with values →0 indicating low population differentiation (low structure - panmixia) and →1 high differentiation (high structure - isolation). F_IS_, the inbreeding coefficient, is the proportion of subpopulation genetic variance contained within one individual’s genotype (i.e. how similar are individuals within subpopulations), with values →1 inferring high inbreeding and →0 low inbreeding.

The target demographic metric was the ‘population growth factor’ λ [37], defined as the ratio of number of individuals at a given time (*N*_*t* + 1_) to that of a previous time (*N*_*t*_). We calculated this as the moving average λ, with *t* from 20 to 200 in 20-year intervals (i.e. *t* =20, 40, 60, … 200). The first 20-year period was excluded as λ was infinite. λ was calculated independently for source and arrival patches. To complement ‘arrival patch λ ’ we also considered the Boolean ‘colonisation success’, defined by the presence of resident individuals in the arrival patch in the year 200. We calculated the demogenetic model outputs described above for each simulation run, setting values for extinct runs to NA (runs that did not reach 200 years). F-statistics were also NA when arrival patch colonisation was unsuccessful (requires a minimum two sub-population structure).

### Regression analyses

We used the machine learning algorithm “randomForest” [50], implemented in the R package “ranger” [51], to analyse and interpret the relationships between the simulation variables and responses. RandomForest (RF) is an efficient procedure that is robust to overfitting and flexible regarding covariate interactions and nonlinearity [50]. Furthermore, RF affords easy and reliable methods for interpretation, which has led to its recent exploitation in individual-based model metamodelling [52].

We first fitted RF metamodels with the demogenetic model outputs (H_o_, F_ST_, F_IS_, ‘source patch λ ’, ‘arrival patch λ ’ and ‘colonisation success’) as dependent variables and the simulation input parameters (‘number of founders’, ‘movement syndrome’, ‘amount of dispersal habitat’, ‘degree of fragmentation’) as independent variables. ‘Colonisation success’ was a classification routine due to its binary nature, while the other continuous outputs were regressions. To understand the underlying processes, we subsequently used RF regression to predict the genetic responses with demographic variables (number residents in “source” patch, number residents in arrival patch, total number of residents, proportion of dispersers changing patch, proportion of dispersers dying, arrival patch λ, and number of successful immigrants [i.e. residence patch ≠ birth patch]) in combination with variables describing the starting conditions, i.e. the simulation input parameters (see above). To interpret all RF models, we computed a permutation based variable importance for regressions, defined as ‘percentage increase of Mean Squared Error’ (%incMSE), and the percentage error rate for classification (i.e. arrival patch colonisation) [51]. We ranked the variables from highest to lowest variable importance. For the RF regression, we visualised the main effects of individual demographic variables and their second-order interaction effects using ‘Accumulated Local Effects’ (ALE) plots, with the R package “ALEplot” [53]. To elucidate the founder and movement syndrome effects, a subset of the simulation parameter space was explored post hoc using pedigree reconstruction, for further methodological details see supplementary material S2.1. We used splines from the R package “mgcv” [54, 55] to interpolate the continuous variables for visualisation of the results (see S2.2).

## Results

**Fig. 3:**
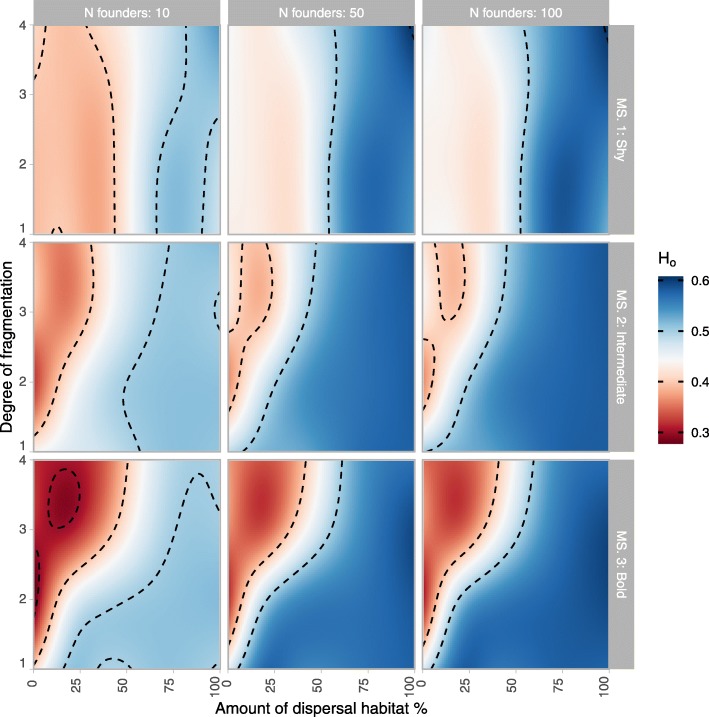
Genetic diversity H_o_ of population in the 200th year after reintroduction. Simulations carried out in a three-patch landscape with “source” and “arrival” breeding habitat patches separated by a “connectivity” patch with varying amounts of ‘dispersal habitat’ and varying ‘degrees of fragmentation’ (i.e. from 1 for randomly distributed dispersal habitat to 4 for large blocks of continuous dispersal habitat), for 3 different movement syndromes (MS 1: shy, MS 2: intermediate, MS 3: bold) and 3 sizes of the founder population

Our simulations showed a nonlinear landscape effect on population genetics (allele frequency) that depended greatly on movement syndrome. The response of genetic diversity (H_o_), across all movement syndromes (MS) and founder population sizes, was twofold (Fig. 3). Increasing the ‘dispersal habitat’ amount from 0 to 100% generally had a positive effect on genetic diversity H_o_. However, for some combinations of ‘movement syndrome’ and ‘number of founders’, there was a lower H_o_ with 10–30% ‘dispersal habitat’ coverage of the connectivity patch compared to 0% ‘dispersal habitat’ coverage (i.e. CS1–100% matrix). As boldness related traits increased (MS1 → MS3), the parameter space domain for which the connectivity landscape had a negative effect on genetic diversity H_o_ shrank and was restricted to more clumped landscapes (degree of fragmentation→4), with lower values of genetic diversity. The extremes in parameter space which had the largest negative and positive effects on genetic diversity were selected for the post-hoc investigation, the results of which are described later.

Increasing the number of founders from 10 to 50 individuals had a positive effect on H_o_, while the effect of increasing from 50 to 100 founders was negligible. This suggests a saturated relationship, constrained by the capacity of the breeding patches. Furthermore, comparing landscapes at the low connectivity extreme of our parameter space, i.e. “connectivity” patch composed of 0% ‘dispersal habitat’ (CS1–100% matrix), to the 100% ‘barrier’ landscape where individuals/genotypes cannot leave the “source” patch (CS3), shows that increasing the number of founders can compensate for genetic losses caused by individuals exploring the ‘matrix habitat’. In other words, the difference in genetic diversity H_o_ between a landscape which readily loses genotypes and a landscape scenario with no lost genotypes tends to zero as the number of founders is increased (Table 2). However, this was also movement syndrome dependent, i.e. the genetic diversity under CS1 and CS3 were equal with more than 50 and 100 founder individuals for MS1 and MS2 respectively, whereas MS3 was not fully compensated by the increases.

**Table 2:** H_o_ across simulated populations in the 200th year after reintroduction. Simulations were carried out for 3 different movement syndromes (shy, intermediate, bold) and 3 sizes of the founder population with “connectivity” patch landscapes: CS1, i.e. 100% matrix – movement out of source patch possible, and CS3, i.e. 100% barrier – movement out of the source patch impossible

N founders	Landscape	MS 1: Shy	MS 2: Intermediate	MS 3: Bold
**10**	CS3	0.42	0.42	0.39
	CS1	0.38	0.36	0.29
**50**	CS3	0.45	0.45	0.44
	CS1	0.44a	0.39	0.35
**100**	CS3	0.46	0.44	0.45
	CS1	0.43a	0.43a	0.33

"a" denotes founder conditions that result in approximately equal H_o_ under CS1 and CS3 (compensation of lost diversity through increased founder population size)

**Fig. 4:**
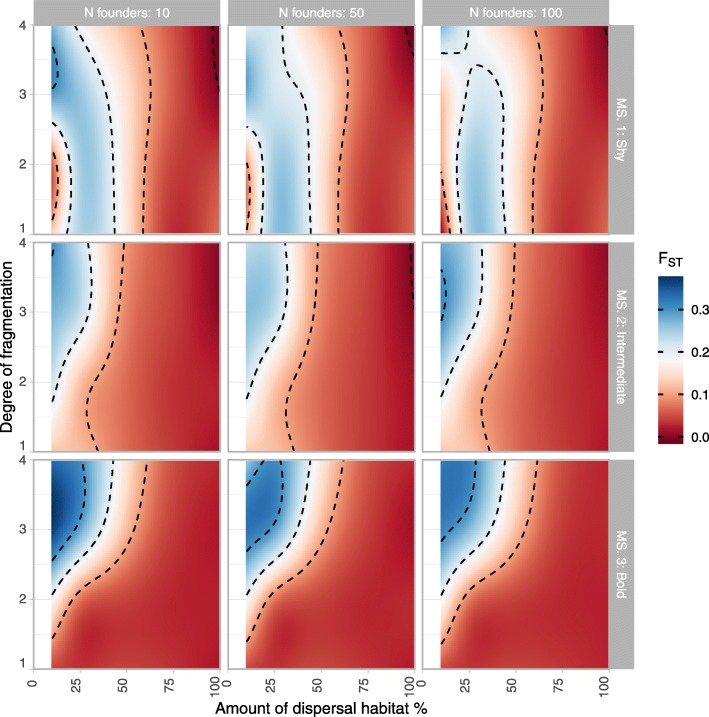
F_ST_ (fixation index - population differentiation due to genetic structure) based on a two sub-population structure in the 200th year after reintroduction. Simulations carried out in a three-patch landscape with “source” and “arrival” breeding habitat patches separated by a “connectivity” patch with varying amounts of ‘dispersal habitat’ and varying ‘degrees of fragmentation’ (i.e. from 1 for randomly distributed dispersal habitat to 4 for large blocks of continuous dispersal habitat), for 3 different movement syndromes (MS 1: shy, MS 2: intermediate, MS 3: bold) and 3 sizes of the founder population

Figure 4 shows the response of F_ST_ - the fixation index (i.e. population differentiation due to genetic structure) was reciprocal to H_o_ such that lower genetic diversity coincided with higher genetic structure, or in other words greater inter-patch differentiation. As such, higher F_ST_ values were seen when the amount of dispersal habitat was between 10 and 50%. In more clumped landscapes (fragmentation→4), high genetic differentiation F_ST_ was only observed if lynx covered larger distances (greater boldness). For metapopulations of endangered species occupying a network of relatively small patches, low population structure is desirable as it suggests greater gene flow between patches.

**Fig. 5:**
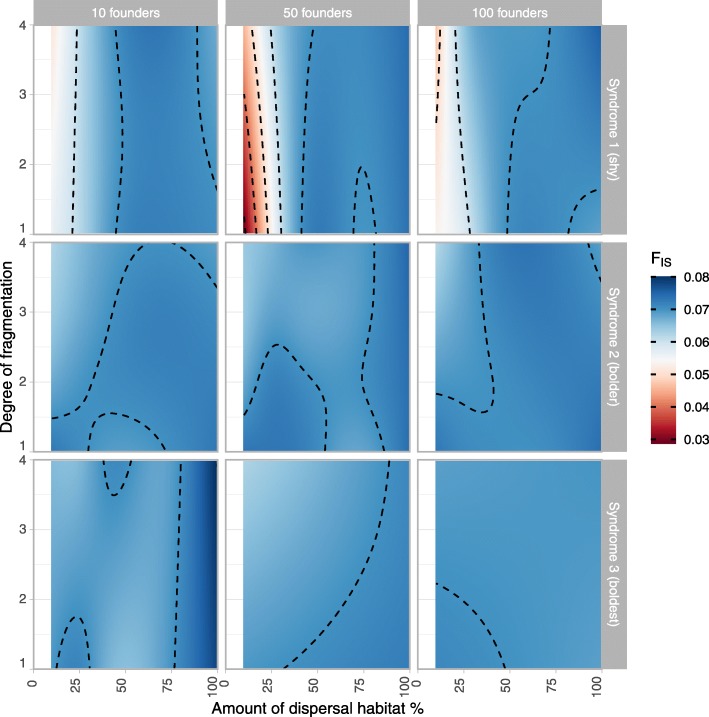
F_IS_ (F-statistic - inbreeding coefficient, proportion of subpopulation genetic variability contained within one individual) based on a two sub-population structure in the 200th year after reintroduction. Simulations carried out in a three-patch landscape with “source” and “arrival” breeding habitat patches separated by a “connectivity” patch with varying amounts of ‘dispersal habitat’ and varying ‘degrees of fragmentation’ (i.e. from 1 for randomly distributed dispersal habitat to 4 for large blocks of continuous dispersal habitat), for 3 different movement syndromes (MS 1: shy, MS 2: intermediate, MS 3: bold) and 3 sizes of the founder population

The inbreeding coefficient F_IS_ (i.e. the proportion of sub-population variance contained within one individual) was lowest in landscapes with low ‘dispersal habitat’ amount (Fig. 5). There was a slight increase of F_IS_ towards higher amounts of ‘dispersal habitat’ in the population with the boldest movement traits (MS.3) and with the smallest number of founders (N founders = 10). Under most landscape conditions F_IS_ was relatively homogeneous, with values between 0.06 to 0.07. In general, the response of F_IS_ to landscape changes was weaker than that of H_o_ and F_ST_.

Genetic metrics seemed to correlate strongly with ‘arrival patch λ’ (Supplementary Material Figure S3), such that low population recruitment in the “arrival” patch is associated with low genetic diversity H_o_, high population differentiation F_ST_ and slightly higher inbreeding F_IS_. In total 298 of 162,810 simulation runs were extinct before year 200. The extinction rate, measured as the fraction of extinct runs for each parameter set, was zero for all simulations with 50 or 100 founder individuals, whereas with 10 founder individuals the average extinction rate was 0.004 for all movement syndromes. The highest extinction rates were in bold populations, with a maximum of 0.017 (Figure S4). Patch connectivity, depicted by the number of individuals changing patches per time step, shows no clear association to genetic diversity (Figure S5). The region of parameter space where higher extinction rates and lower ‘arrival patch λ’ coincided also correlated with low genetic diversity H_o_. Therefore, these regions were investigated post-hoc.

Ranking the variable importance of the RF metamodels showed, the amount of ‘dispersal habitat’ had the highest variable importance for almost all model outputs (F_ST_, F_IS_, ‘source patch λ’ and ‘arrival patch colonisation’). For H_o_ and ‘arrival patch λ’, the number of founders was most important (Table 1). Despite their relatively low rankings, movement syndrome and degree of fragmentation had high importance values for H_o_, F_ST_ and ‘arrival patch colonisation’, indicating their importance in describing functional connectivity.

The results of the RF regression analysis of demographic variables (Table 3) showed that the founder conditions, i.e. movement syndrome and number of founders, play a decisive role for genetic diversity H_o_, and have lower importance for genetic structure (F_ST_, F_IS_). For genetic structure, demographic variables which imply patch connectivity, such as the number of residents in the arrival patch or ‘arrival patch λ’ among others, were more important. Examining the ‘accumulated local effects’ (ALE) plots, we found the positive effects of high connectivity on the continuous independent variables (Figure S6). This was indicated by concurrently increasing H_o_, and decreasing F_ST_ and F_IS_, with increasing migration rate. The effect of number of residents in the arrival patch on H_o_ showed a minimum between 0 and 5 individuals, indicating weak colonisation had a more negative effect on total population genetic diversity than no colonisation.

**Table 3:** Variable importance (%incMSE) and relative ranking (from highest to lowest variable importance) of the demographic and founder condition variables used as predictors in three separate RF regressions with genetic responses (H_o_, F_ST_ and F_IS_)

Independent variables		Demogenetic outputs/dependent variables		
		H_**o**_ {rank}	F_**ST**_ {rank}	F_**IS**_ {rank}
**Demographic variables**	N residents “source” patch	28.45 {8}	17.22 {7}	28.66 {6}
	N residents “arrival” patch	28.72 {7}	**121.91 {1}**	42.61 {4}
	Proportion dispersers changing patches	64.92 {4}	54.61 {4}	57.60 {2}
	Proportion dispersers dying	41.33 {5}	7.12 {8}	20.35 {8}
	“arrival” patch λ	97.25 {3}	95.09 {2}	**79.42 {1}**
	N successful migrants	40.61 {6}	41.84 {5}	52.63 {3}
***Founder conditions***	*Number of founders*	**279.35 {1}**	28.52 {6}	26.75 {7}
	*Movement syndrome*	131.88 {2}	72.70 {3}	40.23 {5}

Our post hoc investigation into a subset of parameter space revealed the influence of founder effects on the population genetic responses. Under landscape conditions with low connectivity, e.g. dispersal habitat amount = 10% and degree of fragmentation = 1 or 4, we found that bolder movement traits (MS1 → MS3) led, on average, to a lower number of founders taking part in reproduction (Figure S7). The combination of greater boldness and clumped ‘dispersal habitat’ in the “connectivity” patch gave rise to a markedly different distribution in the number of descendants per founder, revealing both a high number of founders with few descendants and few founders with many descendants (Figure S8). This contrasts with the less bold movement syndrome (MS1) for which, despite the high number of founders with few descendants, there are fewer ‘dominant founders’ with many descendants. Figure S9 shows that the pattern of lower genetic diversity H_o_, and the movement syndrome/landscape conditions that cause it, coincides with weak colonisation of the arrival patch and leads to higher rates of inbreeding on average.

## Discussion

We extended an existing spatially explicit individual-based population model with neutral genetic markers to: 1) study the combined effects of landscape heterogeneity and various movement syndromes on genetic diversity and structure, exemplified with the novel case of the Eurasian lynx in a Central European landscape, and 2) present this as proof of concept for a demogenetic simulation tool that can provide managers of endangered felid species prognoses before or in the wake of conservation actions. Our study revealed a population level trade off due to the interaction between movement behaviour and landscape. In addition, we were able to delineate the importance of intrinsic and extrinsic factors which drive the underlying process of gene pool dominance. These points, and the biodiversity, species conservation and management context, will be discussed in more detail, along with the study’s limitations, in the following sections.

### General findings and characterisation of ‘risky’ conditions

We identified landscapes with less than 30% dispersal habitat coverage and relatively high clumping in the connectivity patch as “bad” landscape conditions for bold movement behaviour. These conditions led to a higher loss of genetic diversity and an increase in between-population genetic structure. Under most landscape conditions boldness had a positive effect on diversity and decreased population differentiation. Shy movement, on the other hand, generally led to more moderate outcomes, with genetic impoverishment (H_o_ < 0.4) apparent in their “bad” landscapes (less than 50% dispersal habitat coverage) only in scenarios with low founder numbers. The genetic diversity under (movement syndrome-dependent) “bad” landscape conditions was, in some cases, even poorer than in landscapes with zero dispersal habitat or for a closed single patch population. In “bad” landscapes, greater boldness was found to negatively affect the number of reproductive founders and increase genetic dominance of few individuals, due to the fruitless exploration of dispersers, and increase inbreeding at the arrival patch, due to infrequent migration. Therefore, we characterise “bad” landscapes as those which present a tempting proposition for dispersers, but allow only low migration rates. These findings are consistent with the hypothesis of the personality-dependent spatial ecology framework [36], which predicts population level patterns due to interactions between movement syndromes and landscape. Furthermore, our results put us in the position to interpret field findings, which brings new context to empirical monitoring of reintroduced lynx populations. Our approach is a theoretical template for other species, where demogenetic simulations combined with pattern-orientated modelling can be applied to contextualise population viability beyond demographics.

### Importance of intrinsic and extrinsic factors

‘Amount of dispersal habitat’ had high importance for all population demogenetic metrics, underlining functional connectivity’s importance as the main driver of population genetics. The greater importance of dispersal habitat amount over habitat configuration for population genetic structure and diversity is a finding consistent with Jackson et al. [56]. However, in contrast we did not vary breeding habitat amount, as Jackson et al. [56], rather dispersal habitat amount. Dispersal habitat amount was also very important for ‘arrival patch λ’, a key indicator of functional connectivity (a thorough handling of ‘arrival patch λ’ was presented elsewhere [40]). Despite the lower ranks of ‘Degree of fragmentation’ and ‘movement syndrome’ for genetic diversity and genetic structure, their importance was of the same order of magnitude as the higher ranked variables, meaning the metamodel performance suffered similarly with their removal. This reflects the interaction of fragmentation and movement syndrome, whereby boldness increases tolerance to randomly distributed dispersal habitat. ‘Number of founders’ was ranked highest for genetic diversity (H_o_), showing that a high starting diversity can mitigate fixation. Demographic parameters describing conditions in the arrival patch had high predictive importance for genetic structure metrics, whereas “founder conditions” were more important for genetic diversity. A large gene pool is known to affect both genetic diversity and inbreeding coefficient (F_IS_) a priori. Hence, factors affecting genetic diversity, such as the starting gene pool (number of founders), and potential for genetic loss or population growth (both heavily driven by movement syndrome), appeared very important. Contrastingly, the inbreeding coefficient and fixation index (F_ST_) depended on the functional patch connectivity, which was observed in the arrival patch via the emergent number of residents and λ.

### Population-level patterns driven by the movement syndrome trade-off

The trade-off hypothesis [12] posits movement syndrome related fitness trade-offs. For example, bold individuals might take more risks, thereby increasing their mortality, but might have higher reproductive success than shy individuals who survive longer [57]. Although in our study bolder individuals incurred no greater mortality risks (mortality rates were movement syndrome invariant to concentrate on movement driven effects), the interaction of movement syndrome with landscape did reveal reproductive fitness costs. Under certain landscape conditions (fragmentation → 4, dispersal habitat 0–50%), bold movement behaviour resulted in lower genetic diversity than shy movement. In these landscapes, pedigree reconstruction revealed higher rates of genotypic loss and dominance in the bold populations. This was epitomised by the lower number of founder individuals taking part in reproduction, a high number of individuals with few, or zero, descendants, and higher inbreeding, especially in the arrival patch, due to reproductive dominance of few individuals. When functional connectivity was low, the few females that managed to colonise the arrival patch were dominated by even fewer ‘super males’ (territorialism parametrised with 1 male to 1–3 females). In addition, the higher propensity for bold populations to explore the matrix left smaller founder populations in the source patch. Conversely, the shy populations maintained a stable number of reproductive founders, thereby reducing the loss and dominance, and minimising inbreeding. The movement syndrome trade-off exists when the poor outcome thanks to boldness is compared to the higher genetic diversity boldness fostered under most other landscape conditions. Here, greater genetic exchange between patches was accompanied by a relatively high proportion of founders participating in reproduction and similar inbreeding rates in both “source” and “arrival” patches. Although we only simulated monomorphic populations (i.e. movement syndromes varied between runs), the results suggest that a diversity in movement syndromes might buffer this trade-off and could possibly lead to optimal outcomes across various landscapes.

Domination of the gene pool by successful founders in the arrival patch, with the by-product of lower genetic diversity, is consistent with the principle of high-density blocking where the ‘founder takes all’ [58]. This terminology describes situations where the first individuals to arrive and reproduce in previously unoccupied territories dominate the gene pool (founder takes all) and thus reduce genetic diversity by excluding other individuals (blocking). This has been implicated in colonisation events following glaciation [59] and human driven extinctions [60]. Although in our experiment all movement syndromes display this phenomenon, the effect is more pronounced for bolder movement. This is driven by their higher propensity to explore the landscape under conditions of poor patch connectivity, which even creates a ‘founder takes all’ effect in the source patch. Furthermore, increasing the number of founders did not fully compensate the ‘founder takes all’ effect observed. This inextricably links conservation management to the spatial behaviour of animals and highlights the need to consider target patches within their habitat networks.

### Felid conservation management

In general, we showed that increasing the number of founders in a reintroduced population is beneficial for genetic diversity and would compensate for genotypic losses (exploring/dying dispersers) or inbreeding due to dominance in weakly colonised patches. In contrast to our experiments, in reality some founder relatedness is unavoidable, therefore compensation of these phenomena would require more founders. The importance of introducing enough founders to buffer stochastic effects of small populations and genetic bottlenecks due to Allee effects and drift [61] is known a priori. Despite this, insufficient founder populations has been identified as one of the major problems in reintroduction programmes [62]. The common ‘rule of thumb’ recommends releasing as many individuals as necessary to reach at least 20–30 breeding individuals [63]. To fulfil this, our results suggest that the wider landscape context should be considered when selecting reintroduction patches. A network of patches in the vicinity of a reintroduction could, in the worst-case, divide the gene pool and allow dominance of few individuals. Recently, a reintroduction project recaptured and relocated a translocated lynx to avoid losing this individual to the target area [64]. Such interventions, or more founders, could mitigate potential losses when the target patch is situated in a poorly connected network.

In our simulations the initial genetic diversity (H_o_) is extremely high, however, after 200 years (approx. 40 generations) it reached levels that could be expected from real populations with less diverse origins only a few decades after reintroduction (H_o_≈ 0.3 to 0.6 (e.g. [34, 65, 66]). Under favourable landscape conditions and with 50 or more founders, H_o_ was as high as in autochtonous populations (e.g. [67]). Even with 10 founders H_o_ > 0.5 was possible, which given the higher founder numbers in contemporary reintroductions, albeit with smaller initial gene pools, might offer hope for the longevity of these and future reintroduction efforts. However, if we consider a reintroduced population in Central Europe (e.g. Bavarian-Bohemian-Austrian or Harz Mountains) as a potential “source” patch and a suitable unoccupied “arrival” patch (e.g. Thuringian Forest), with the landscape of Germany in between as a “connectivity” patch (i.e. forest cover ≈ 30% and moderate habitat fragmentation [68]), a subsequently connected population including two patches under our conditions might expect a total population H_o_ between 0.35 and 0.47, F_ST_ between 0.5 and 0.2 and F_IS_ between 0.05 and 0.08 – depending on the composition of movement syndromes. These values represent, at best, a poor genetic diversity comparable to some reintroduced lynx populations [34], a significant population differentiation [69] and a level of inbreeding that motivated the contemporary population reinforcement of the Dinaric lynx population [70].

### Limitations of our study

Some limitations must be recognised in our study. Firstly, animal syndromes do not exist in just the shy-bold axis [10], and they are believed to exist in correlated trait axes (i.e. bold-exploratory-aggressive or shy-thorough-sociable types) [11]. Since movement traits which imply boldness are not necessarily associated with other life-history events [71, 72], we decided to simplify matters and neglect other supposedly correlated traits (or demographic rates), allowing less complex inference. Unfortunately, empirical research on felid syndromes does not yet extend to wild living felids [73]. Personalities have, however, been shown for various felid species in captivity, including Snow leopard (*Uncia uncia*) [74], Clouded leopard (*Neofelis nebulosa*) [75] and Bengal tiger (*Panthera tigris tigris*) [76]. In terms of spatial ecology, so far there have been only cautious inferences for domestic [77] and feral [78] cats (*Felis catus*) regarding dominant/subordinate personalities and home range sizes. Despite this, the growing pool of research that relates variability in movement behaviour to personality motivated our assumptions. For example, the existence of fast/slow, social/solitary and shy/bold behavioural syndromes have been shown to affect, respectively, dispersal distance [79], dispersal propensity [80] and distance moved [81]. Furthermore, the combination of high variability in spatial behaviour of felids (e.g. dispersal distances of jaguar [82] or lynx [83] and home range size of leopards [84] or lynx [85]) and limited samples common with telemetry-based research [86], suggests the importance of behavioural syndromes could be found if studies had sufficient power [87]. Using different parameterisations, we could evaluate a range of scenarios and identify potential trade-offs between movement behaviour and population level genetic metrics. Furthermore, to disentangle the influences of the different factors we chose to simulate movement syndromes discretely (monomorphic populations). We applied this method to avoid assumptions of heritability, whether genetic or environmental, in movement syndromes.

Secondly, we used a simple three-patch system with two breeding habitat patches separated by a larger dispersal and matrix habitat patch and neglected movement syndrome-dependent territory size, although this has been shown for some species (e.g. [88]). Under these conditions we could keep the breeding habitat constant, thereby avoiding geometric considerations, such as placement of territories in fragmented habitats, and keep population size approximately constant. These simplifications helped us eliminate many confounding factors and focus our attention on understanding the potential effects of movement behaviour on (lynx) population genetics.

Thirdly, in this study we respectively simplified and neglected the two main mortality factors found in Europe, e.g. road traffic collisions and illegal hunting [38, 89–91]. We accounted for the difference in road mortality between residents and dispersers by assuming higher natural mortality for dispersers than residents and neglected a spatially explicit road mortality model. We also did not consider mortality differences between matrix and dispersal habitat, as this was not supported by empirical studies [37]. Furthermore, this would have only modified which landscapes promoted the ‘founder takes all’ effect, since greater mortality of dispersers is functionally equivalent to reducing the number of founders. In addition, we did not consider negative effects of inbreeding on fitness or fecundity. There is evidence that inbreeding and low genetic diversity can affect fitness. For example, hereditary heart defects in a Swiss lynx population and the Dinaric Mountains lynx population crash are thought to be related to inbreeding and genetic impoverishment [70, 92]. Removing these fitness-reducing factors leads to higher overall estimates of genetic diversity and structure, since genotypes are not as rapidly lost.

### Future perspectives for research and conservation

As the stability and resilience of reintroduced populations depends heavily on movement traits, future work should consider the existence of intraspecific trait variability in wild felid populations. As a first step for lynx, empirical analyses of movement behaviour, which have mainly been focused on natal dispersal distances and times (e.g. [93–95]), could be deepened. An individual-based mechanistic movement model was first developed using radio telemetry [41]. Since this time GPS technology has flourished, resulting in the extensive collection of telemetry data from collared animals and development of new techniques for movement analysis (e.g. [96]). A contemporary reanalysis with higher spatiotemporal resolution could account for more detailed movement processes, which would progress lynx movement ecology research, and could form the basis of future demogenetic simulation studies.

Utilising such empirical information, demogenetic simulations could explicitly consider movement behaviour diversity and better constrain predicted population viability and long-term genetic diversity for reintroductions and reinforcement projects. The demogenetic model could, for example, help clarify the relationships between founder population size or sex ratio, demographic rates (e.g. breeding success) and the genetic diversity of reintroduced populations. In particular, since rules of thumb like the “one migrant per generation” rule have come under scrutiny due to their theoretical nature [97, 98], it would be useful to develop some species, or even population, specific recommendations that take into account individual processes such as movement behaviour. Here, further empirical research combined with our approach could directly support management.

## Conclusions

There is increasing support being voiced for the value of individual differences at various levels of organisation [10], from species [99] to ecosystem functioning [100]. We stress the importance of behavioural syndromes, in this case, for the genetics of reintroduced lynx populations. We found bold-explorers could be saviours - by connecting populations and fostering panmixia, or sinks – by readily exemplifying the ‘founder takes all’ principle under unfavourable landscape conditions. In their absence shy-stayers might slowly succumb to genetic drift and/or inbreeding.

Populations of endangered species are becoming ever more restricted in size and mobility, leaving them at risk. Movement traits could be crucial for establishing exchange between subpopulations and deciding the fate of at-risk species. Management needs to be informed of the hazards and opportunities, not only regarding extinction risk but also long-term viability, hence genetic diversity.

Demogenetic simulations based on empirical field data have potential to deliver recommendations for conservation management, including population reintroductions and reinforcement projects. Here we presented the first demogenetic model for felids that includes spatially explicit movement, neutral genetic markers, realistic reproduction and inheritance processes (i.e. territory searching, occupation, overlap and mating). Such simulation tools can be applied to various at risk or endangered species, here exemplified by Eurasian lynx. In the future, this model could be used to assess the consequences of movement traits, landscape conditions and mortality on demographic viability and genetic potential under diverse scenarios. Future research should deliver prognoses and recommendations under specific management scenarios that could: 1) reinforce genetic diversity of felid populations to reach target levels, and 2) maintain target levels of genetic diversity in meta-populations. Finally, given the crucial role movement plays in population persistence, simulation studies should aim to help close knowledge gaps identified for various felid species [101].

## Supplementary information

**Additional file 1. ODD Protocol –** Description of “The Eurasian Lynx Dispersal, Demographic and Genetic Model”.

**Additional file 2: Additional methods: S2.1** Post hoc investigation of pedigree structure, and **S2.2** Interpolation of the continuous variables using smooth splines for visualisation.

**Additional file 3: Figure S3.** “Arrival” patch λ, based on a two sub-populations structure in the 200th year after reintroduction. Simulations were carried out in neutral landscapes of varying amounts of dispersal habitat and varying degrees of fragmentation (i.e. from 1 for randomly distributed dispersal habitat to 4 for large blocks of continuous dispersal habitat), for 3 movement syndromes (MS 1: shy, MS 2: intermediate, MS 3: bold) and 3 sizes of the founder population.

**Additional file 4: Figure S4.** Simulation extinction rates for all of parameter space.

**Additional file 5: Figure S5.** Number of disperser individuals changing breeding patch in the 200th year as a proxy for connectivity, by gender and with genetic diversity H_o_.

**Additional file 6: Figure S6.** Accumulated Local Effects (ALE) plots for RF regression analysis of genetic outputs H_o_, F_ST_ and F_IS_, using demographic covariates.

**Additional file 7: Figure S7.** Number of founder individuals taking part in reproduction by gender, for all simulation runs of a subset of parameter space (see S2.1). For A – 10 founders, and B – 50 founders. The mean values indicated by the dotted line.

**Additional file 8: Figure S8.** Density distribution of total number of descendants of founder individuals 50 years after reintroduction, by gender, for all simulation runs of a subset of parameter space (see S2.1). For A – 10 founders, and B – 50 founders.

**Additional file 9: Figure S9.** Individual inbreeding values of living individuals 50 years after reintroduction, by patch (1 = “source”, 2 = “arrival”), for all simulation runs of a subset of parameter space (see S2.1). For A – 10 founders and B – 50 founders. Inbreeding calculated by pedigree construction. Mean Inbreeding indicated by red line.

## Data Availability

The datasets used and/or analysed during the current study are available from the corresponding author on reasonable request. The simulation tool is also available from the authors on request as a Windows desktop application. It will be publicly available online soon.

## Abbreviations

MS: Movement syndrome
ALE: Accumulated Local Effects
CS: Control scenarios
ODD: Overview, Design concepts, and Details protocol
RF: Random Forest
SEDM: Spatially Explicit Dispersal Model
SEIBM: Spatially Explicit Individual-Based Model
SMM: Stepwise Mutation Model
%incMSE: Percentage increase of Mean Squared Error
