# Supplementary material for: The boon and bane of boldness: movement syndrome as saviour and sink for population genetic diversity
Source: Mov Ecol. 2020 Apr 21;8:16. doi: 10.1186/s40462-020-00204-y (PMC7175569; doi:10.1186/s40462-020-00204-y)
Supplement: Supplementary file 1 — Additional file 1. ODD Protocol – Description of “The Eurasian Lynx Dispersal, Demographic and Genetic Model”. [file 40462_2020_204_MOESM1_ESM.docx]

**The Eurasian Lynx Dispersal, Demographic and Genetic Model**

We describe the model following the ODD protocol for individual-based models (Overview, Design Concepts, Detail; Grimm et al. 2010; Grimm et al. 2006; Grimm and Railsback 2005). This ODD is protocol is based on that of Kramer-Schadt et al. (2011) with changes to reflect the new sub-model.

**Purpose**

We developed an individual-based, spatially-explicit population simulation model with neutral genetic markers, or - a demogenetic population model. This was used to assess the development of genetic structure and diversity of reintroduced lynx populations in heterogeneous landscapes under scenarios of different movement syndromes and founder population sizes. The original model which did not incorporate neutral genetic markers was previously published in a number of papers where it was used for a variety of purposes, e.g. for assessing the additional impact of roads (Klar et al. 2006), perceptual range (Pe'er and Kramer-Schadt 2008), stepping stones (Kramer-Schadt et al. 2011), or unknown mortality (Heurich et al. 2018) on population connectivity and viability. Here, we describe the basic model common to the current and preceding papers, plus the additional genetic sub-model; specific settings, simulation experiments and changes to the model rules are described in the respective publications.

**State variables and scales**

The model consists of four sub-models, 1) a demographic model of lynx considering territory occupation, reproduction, and mortality, 2) a dispersal model that links the demographic processes onto a landscape sub-model (3), and 4) a genetic sub-model that handles neutral genetic markers. Demographic parameters stem from published data of long-term field studies in fragmented landscapes in Switzerland, Poland and Spain (Ferreras et al. 1992; Jedrzejewski et al. 1996; Breitenmoser-Würsten et al. 2001; Schmidt-Posthaus et al. 2002). The original dispersal module (Kramer-Schadt et al. 2004) was calibrated with field data from dispersing lynx collected in the Swiss Jura Mountains (Breitenmoser et al. 1993).

The state variables of the lynx individuals are (1) sex, (2) age, (3) location (x-y coordinates), (4) demographic status (disperser or resident) and (5) neutral genetic markers (microsatellite loci lengths). The landscape consists of a grid of 1 km^2^ cells which corresponds with the animal’s perceptual range as the smallest spatial unit. Cells represent functional landscape types for lynx and are classed in (a) suitable for breeding, (b) suitable for dispersal, (c) avoided, but used occasionally, and (d) barriers (Schadt et al. 2002). Landscape borders are reflective. Time steps represent one day to capture the variability in daily dispersal distance in concert with the landscape variability. Simulation time (in years) is variable and depends on the question addressed.

**Process overview and scheduling**

At the beginning of each model time step (year), the number of resident and non-resident males and females on the landscape map are determined. All non-residents older than 1 year disperse and search for territories (see Dispersal Sub-model). Each day, non-resident individuals disperse a certain number of steps with the direction depending on the underlying landscape type, search for a territory and undergo a daily mortality probability. The spatially explicit processes of dispersal and territory selection in the model depend upon local habitat quality within the immediate surrounding of the animals’ location. If dispersing individuals survive (probabilistic event depending on daily dispersal mortality *M_disp_*), they settle or continue dispersing in the following year. At the end of each daily time step, the location of the dispersing individuals is updated. If the disperser has found and occupied a territory, the status is set to resident. Next, the demographic, and therefore also genetic, processes of the residents come into play (see Demographic Sub-model). At the end of each year, the occupied territories of the residents and the age and status of each lynx are updated (see Fig. ODD1).

**Design concepts**

The model considers lynx demography and genetic inheritance, dispersal, territory selection and occupation and interaction of the landscape types with these ecological processes. The behavior and demography of the lynx are imposed by reaction towards the landscape types as well as by status-dependent parameters. Stochasticity is included to represent demographic and environmental noise. Allee effects are considered in the demographic sub-model: only when male and female lynx have overlapping territories is reproduction considered. Inheritance of neutral genetic markers is determined by Mendelian inheritance and is inherently stochastic. Genetic mutation during reproduction is considered in the genetic sub-model such that allele loci may mutate via a stepwise mutation model at a given rate.

**Initialization**

Initial population characteristics (size, age distribution, location, population genetics) are variable and are set via individuals’ state variables. Each individual is attributed: sex, location (x-y coordinates), and 12 loci-pairs (i.e. 24 neutral genetic markers).

**Input**

The model does not include any external model or data files of driving environmental variables.

**Sub-models**

*Demographic sub-model.*— This sub-model controls the individuals with resident status. Territory occupation, reproduction, and resident mortality are processes happening on an annual level. Each resident female whose territory is overlapped by that of a male reproduces with a certain probability (*P_birth_*, Table T1). We set the probability of having one or two cubs surviving their first year to 0.5 and the sex ratio to 1:1 Annual resident mortality (*M_res_*) is also a probabilistic event. The cells belonging to a resident’s territory are kept if the individual survives.

*Genetic sub-model. —* This sub-model handles the diploid genotypes (neutral genetic markers modelled as microsatellite loci lengths) during reproduction events. Each cub which survives their first year inherits 1 maternal and 1 paternal allele at each loci (total 12 loci allele pairs), with an even probability given to each parental allele. Genetic mutation is simulated using a stepwise mutation model (SMM). During reproduction the inherited alleles undergo mutation at a chosen rate. If mutation of an allele takes place the SMM is applied; as such an integer allele A_0_ has an even probability of increasing or decreasing its value by 1, to A_0_+1 or A_0_-1. The lower limit is constrained as a microsatellite length of zero is not possible, hence if A_0_=1 a length decreasing step is not permitted and an even probability is assumed for either increasing or keeping the allele length.

*Dispersal sub-model (including territory searching behaviour).—* Each day, a certain number of movement steps (*s*) is assigned, based on model calibration with field data (Table T1). The spatial unit of dispersal is one movement step, i.e. 1 km^2^ grid cell. In each step, individuals survey their eight-cell neighbourhood and make decisions based on this information (see below). Their choice of direction is comprised of two components: the probability of leaving preferred dispersal habitat by stepping into the matrix (*P_matrix_*, Table T1), and a correlation factor determining the probability of continuing with the same direction as their previous movement within a day (*P_C_*, Table T1). The hierarchy is a preference of dispersal habitat over a persistent movement forward, with the first direction of every day chosen randomly. Within a day, the next cell is chosen based on the preference for dispersal habitat and the avoidance of matrix. If the neighbourhood of a dispersing lynx, comprising the origin cell and its 8 neighbours, contains only matrix or dispersal habitat cells, the probability of choosing one of these cells is random (i.e., 1/9). However, if the neighbourhood is a mixture of dispersal habitat and matrix, we consider the preference for dispersal habitat as follows: The number of matrix cells *n_mat_* within the neighbourhood is counted. The probability of leaving dispersal habitat *P_leave_* is then dependent on the number of matrix cells around the origin cell multiplied by a factor *P_matrix_* (ranging from total avoidance of matrix [*P_matrix_* = 0] to randomly choosing any surrounding cell [*P_matrix_* = 1/9], with *P_leave_* = *n_mat_* * *P_matrix_*. If an animal has stepped into the matrix, it is assigned a ‘memory’ of its last location in a dispersal habitat, toward which it returns should it fail to find a dispersal habitat cell within *P_maxmatrix_* = 10 steps. A daily mortality probability is included (*M_disp_*, Table T1). We note that the mortality probability *M_disp_* is landscape-independent due to the absence of sufficient field-data regarding mortality risks in different landscape types.

We upscale the landscape in terms of territory searching behaviour, i.e., each dispersing female needs to collect a certain amount of contagious cells of non-occupied breeding habitat (*N_HRCells_*), whereas males search for cells that are already occupied by females and can overlap up to 3 females. To include stochasticity in territory size we draw for each female a random number of cells from a uniform distribution *N_HRCells_* between 70 and 100 cells. The simulated female then has to use this amount of cells as her territory. Once occupied, territory cells cannot be used by other females. In unoccupied areas the female that comes first has the best chance of occupying a territory.

**Tab et al., 2005)**

**Table T1.** Basic parameter values for the demographic and the dispersal sub-models. Values can change depending on the scenarios assessed in the respective publications.

| **Sub-model** | **Symbol** | **Parameter value or range** |
| --- | --- | --- |
| *Demographic sub-model* |  |  |
| Reproduction rate (=prob. of giving birth) | *P*_birth_ | 0.75 |
| Annual mortality probability of residents | *M*_res_ | 0.1 (translates into ~13% annual mortality rate) |
| *Genetic sub-model* |  |  |
| Mutation rate |  | 10^-4^/locus |
| *Dispersal sub-model* |  |  |
| Correlation factor | *P*_C_ | 0.5 |
| Probability of stepping into matrix | *P*_matrix_ | 0.03 |
| Maximum number of steps an individual stays in matrix before returning | *P_maxmatrix_* | 10 |
| Maximum number of steps per day | *s*_max_ | 45 |
| Exponent of step distribution | *x* | 11 |
| Daily mortality probability of dispersers | *M*_disp_ | 0.0007 (translates into ~22% annual mortality rate) |

Each day, each dispersing lynx is assigned a certain number of movement steps *s* based on a probability *P*(*s*) using a power function with an exponent *x* and parameter *s_max_* that determines the maximum number of steps that a dispersing lynx can cover during a single day:

*P(s)* = (1 − ((*s* − 1)/(*s_max_* − 1)))^x^.


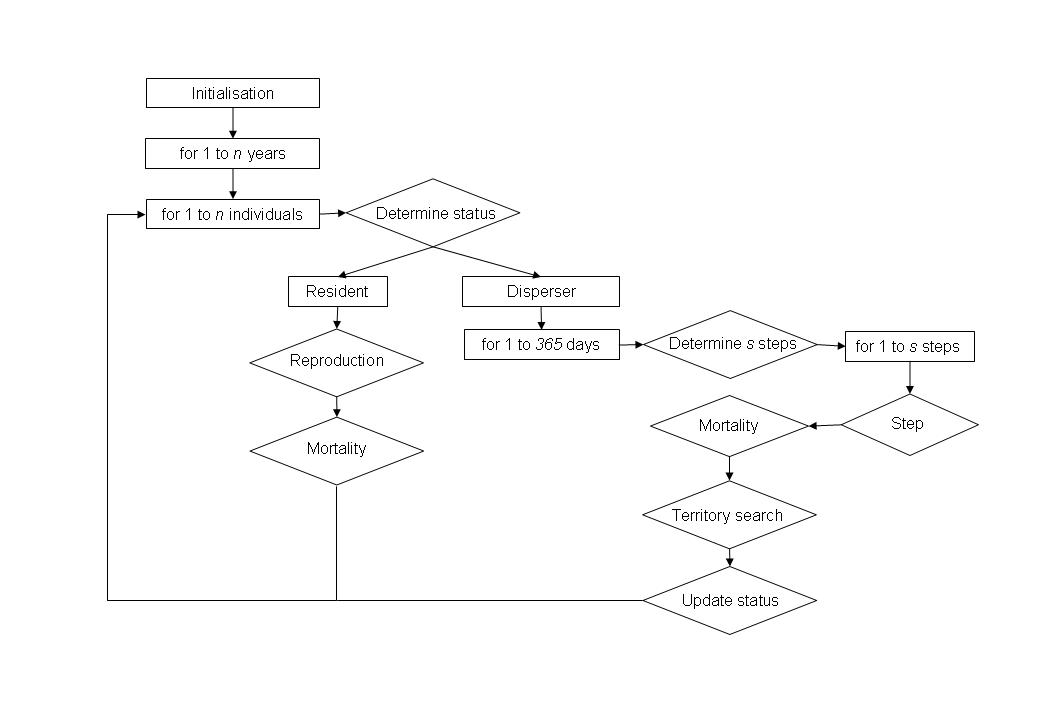


**Figure ODD1.** Flow chart of the model steps.

**References**

Breitenmoser U., Kaczensky P., Dötterer M., Breitenmoser-Würsten C., Capt S., Bernhart F. and Liberek M. 1993. Spatial organization and recruitment of lynx (*Lynx lynx*) in a re-introduced population in the Swiss Jura Mountains. Journal of Zoology (London) 231: 449-464.

Breitenmoser-Würsten C., Zimmermann F., Ryser A., Capt S., Laass S., Siegenthaler A. and Breitenmoser U. 2001. Untersuchungen zur Luchspopulation in den Nordwestalpen der Schweiz 1997-2000. KORA Bericht 9: 92.

Ferreras P., Aldama J.J., Beltran J.F. and Delibes M. 1992. Rates and causes of mortality in a fragmented population of Iberian lynx (*Felis pardina* Temminck, 1824). Biological Conservation 61: 197-202.

Grimm V., Berger U., DeAngelis D.L., Polhill J.G., Giske J. and Railsback S.F. 2010. The ODD protocol: A review and first update. Ecological Modelling 221: 2760-2768.

Grimm V., Berger U., Bastiansen F., Eliassen S., Ginot V., Giske J., Goss-Custard J., Grand T., Heinz S.K., Huse G., Huth A., Jepsen J.U., Jorgensen C., Mooij W.M., Müller B., Pe'er G., Piou C., Railsback S.F., Robbins A.M., Robbins M.M., Rossmanith E., Rüger N., Strand E., Souissi S., Stillman R.A., Vabo R., Visser U. and DeAngelis D.L. 2006. A standard protocol for describing individual-based and agent-based models. Ecological Modelling 198: 115-126.

Grimm V. and Railsback S.F. 2005. Individual-based modeling and ecology, Princeton University Press, Princeton N.J.

Jedrzejewski W., Jedrzejewska B., Okarma H., Schmidt K., Bunevich A.N. and Milkowski L. 1996. Population dynamics (1869-1994), demography, and home ranges of the lynx in Bialowieza Primeval Forest (Poland and Belarus). Ecography 19: 122-138.

Klar N., Hermann M. and Kramer-Schadt S. 2006. Effects of roads on a founder population of lynx in the biosphere reserve 'Pfälzerwald - Vosges du Nord'. A model as planning tool. Naturschutz und Landschaftsplanung 38: 330-337.

Kramer-Schadt S., Revilla E. and Wiegand T. 2005. Lynx reintroductions in fragmented landscapes of Germany: projects with a future or misunderstood wildlife conservation? Biological Conservation 125: 169-182.

Kramer-Schadt S., Revilla E., Wiegand T. and Breitenmoser U. 2004. Fragmented landscapes, road mortality and patch connectivity: modelling influences on the dispersal of Eurasian lynx. Journal of Applied Ecology 41: 711-723.

Pe'er G. and Kramer-Schadt S. 2008. Incorporating the perceptual range of animals into connectivity models. Ecological Modelling 213: 73-85.

Schadt S., Revilla E., Wiegand T., Knauer F., Kaczensky P., Breitenmoser U., Bufka L., Cerveny J., Koubek P., Huber T., Stanisa C. and Trepl L. 2002. Assessing the suitability of central European landscapes for the reintroduction of Eurasian lynx. Journal of Applied Ecology 39: 189-203.

Schmidt-Posthaus H., Breitenmoser-Würsten C., Posthaus H., Bacciarini L. and Breitenmoser U. 2002. Causes of mortality in reintroduced Eurasian lynx in Switzerland. Journal of Wildlife Diseases 38: 84-92.
