# Supplementary material for: The boon and bane of boldness: movement syndrome as saviour and sink for population genetic diversity
Source: Mov Ecol. 2020 Apr 21;8:16. doi: 10.1186/s40462-020-00204-y (PMC7175569; doi:10.1186/s40462-020-00204-y)
Supplement: Supplementary file 2 — Additional file 2: Additional methods: S2.1 Post hoc investigation of pedigree structure, and S2.2 Interpolation of the continuous variables using smooth splines for visualisation. [file 40462_2020_204_MOESM2_ESM.docx]

# Additional material S2 – additional methods

## S2.1 - Post hoc investigation of pedigree structure

In addition to the simulations described in the section *Methods, Model outputs,* we also conducted simulation runs post hoc using a subset of parameter space to investigate pedigree structure. This was motivated by the regression analysis results which could not attribute the genetic outcomes satisfactorily to changes in demographic processes caused by the interaction of landscape structure and movement syndromes. The parameter space subset was selected based on visual inspection of the results (main text Fig. 3, 4, 5). The parameter values identified (S2 Table 1) define the extremes of genetic diversity ($H_{o}$) in the main simulation experiments. For comparison, simulations with control landscapes were also conducted (only ‘dispersal habitat’, ‘matrix habitat’ or ‘barrier’ in the “connectivity” patch).

| **Subset** | **Number of founders** | **Movement syndrome** | **Amount of dispersal habitat** | **Degree of fragmentation** |
| --- | --- | --- | --- | --- |
| 1 | 10 | 1 | 10 | 1 |
| 2 |  |  | 10 | 4 |
| 3 |  |  | 80 | 4 |
| 4 |  | 3 | 10 | 1 |
| 5 |  |  | 10 | 4 |
| 6 |  |  | 80 | 4 |
| 7 | 50 | 1 | 10 | 1 |
| 8 |  |  | 10 | 4 |
| 9 |  |  | 80 | 4 |
| 10 |  | 3 | 10 | 1 |
| 11 |  |  | 10 | 4 |
| 12 |  |  | 80 | 4 |

***S2 table 1*** Summary of reintroduction and landscape structure parameters selected for the post hoc investigations.

## Simulations and model outputs

In broad terms the simulation experiments followed those described in the main text. In contrast, these simulation experiments were conducted only for 50 years with model outputs recorded for every time step (yearly) instead of 20-year intervals. In addition, we used the individual IDs with the maternal and paternal IDs to reconstruct the pedigree and inbreeding of the populations at given timesteps. Pedigree and inbreeding were calculated using the R package “pedigree” [1] and the following results were plotted: (i) total number of descendants per founder by year 50, (ii) the number of founders taking part in reproduction (number of offspring>1) and (iii) the mean inbreeding of living individuals at year 50.

## S2.2 – Interpolation of the continuous variables using smooth splines

For visualisation of the simulation experiment results interpolation was carried out using smooth splines from the R package “mgcv” [2,3]. The independent covariates ‘dispersal habitat amount’ and ‘habitat fragmentation’ were fitted to the dependent variables ($H_{o}$, $F_{\mathrm{ST}}$, $F_{\mathrm{IS}}$ and arrival patch λ) using tensor products (2-dimensional anisotropic splines) grouped by ‘movement syndrome’ and ‘founder population size’ with knots k=[5,4] and the form:

*Dependent variable ~ 1 + founders*syndrome + te(amount, fragmentation, by=founders:syndrome)*

## References

1. Coster A, Coster MA. Package ‘pedigree’. R package version. 2010 Jun 21;1.

2. Wood SN. Generalized additive models: an introduction with R. Chapman and Hall/CRC; 2017.

3. Wood SN. Fast stable restricted maximum likelihood and marginal likelihood estimation of semiparametric generalized linear models. Journal of the Royal Statistical Society Series B (Statistical Methodology). 2011;73(1):3–36
