# Supplementary figures and images for: The boon and bane of boldness: movement syndrome as saviour and sink for population genetic diversity
Source: Mov Ecol. 2020 Apr 21;8:16. doi: 10.1186/s40462-020-00204-y (PMC7175569; doi:10.1186/s40462-020-00204-y)

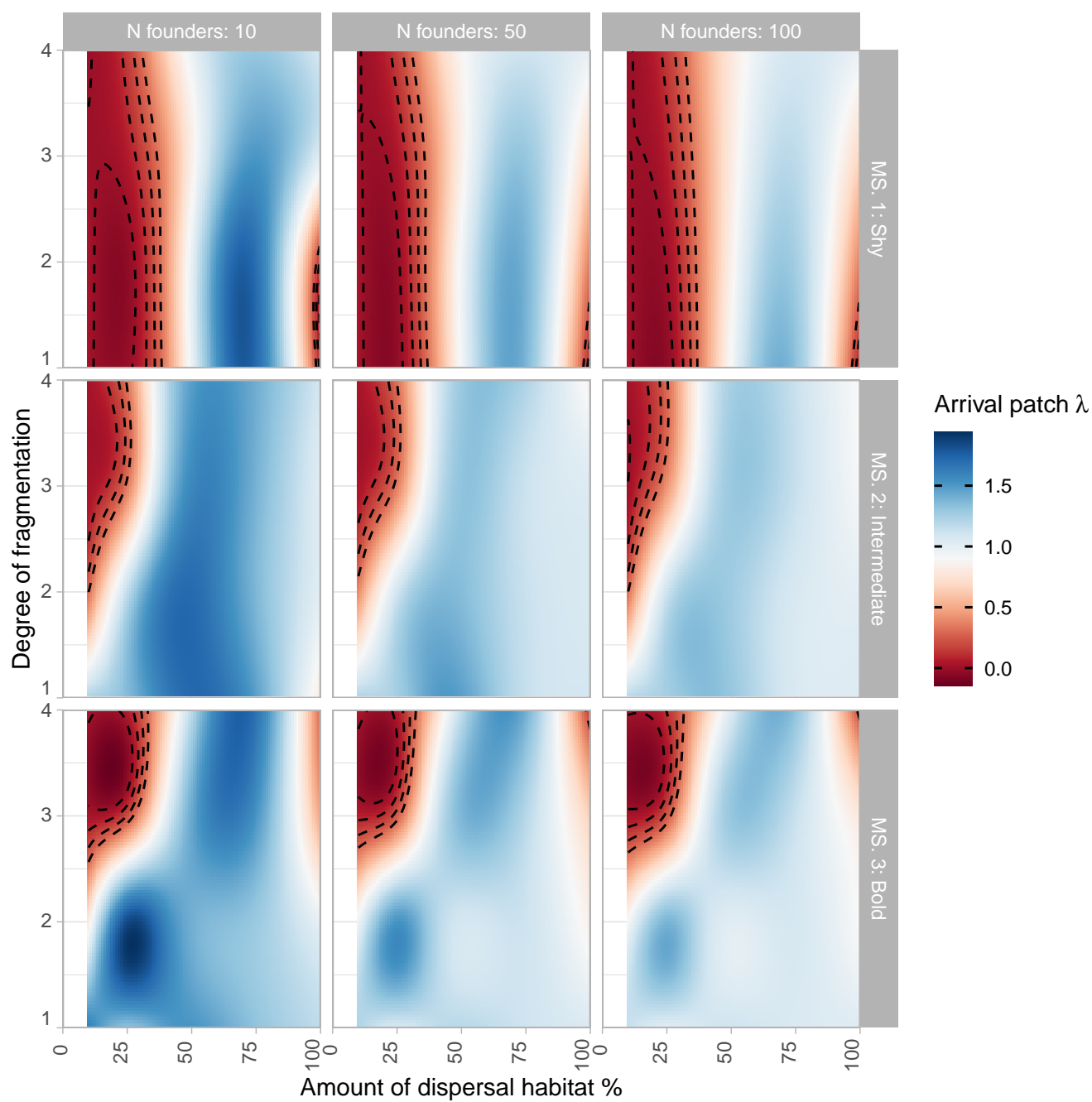

Supplement: Supplementary file 3 — Additional file 3: Figure S3. “Arrival” patch λ, based on a two sub-populations structure in the 200th year after reintroduction. Simulations were carried out in neutral landscapes of varying amounts of dispersal habitat and varying degrees of fragmentation (i.e. from 1 for randomly distributed dispersal habitat to 4 for large blocks of continuous dispersal habitat), for 3 movement syndromes (MS 1: shy, MS 2: intermediate, MS 3: bold) and 3 sizes of the founder population. [file 40462_2020_204_MOESM3_ESM.pdf]

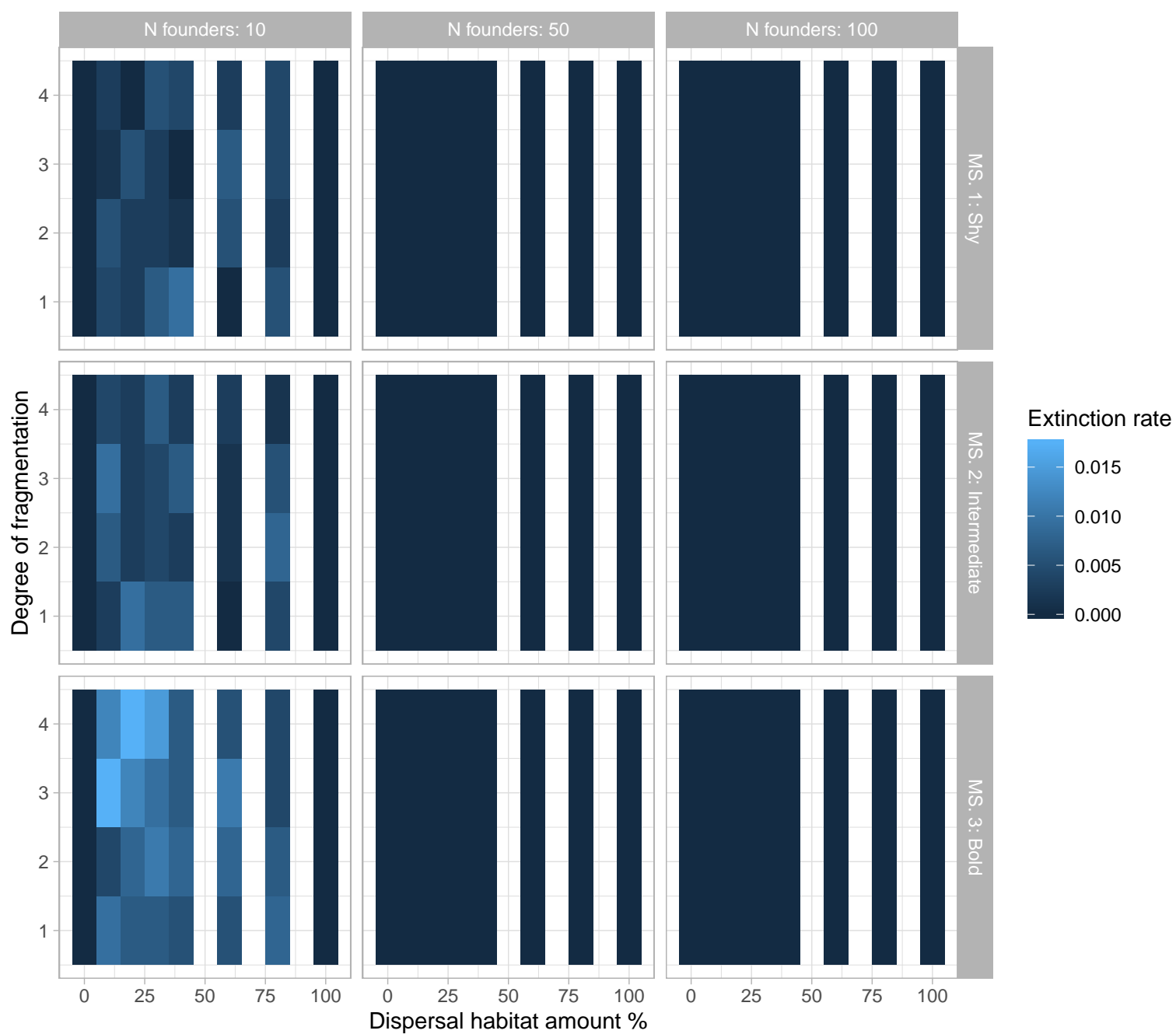

Supplement: Supplementary file 4 — Additional file 4: Figure S4. Simulation extinction rates for all of parameter space. [file 40462_2020_204_MOESM4_ESM.pdf]

## A: All with Ho

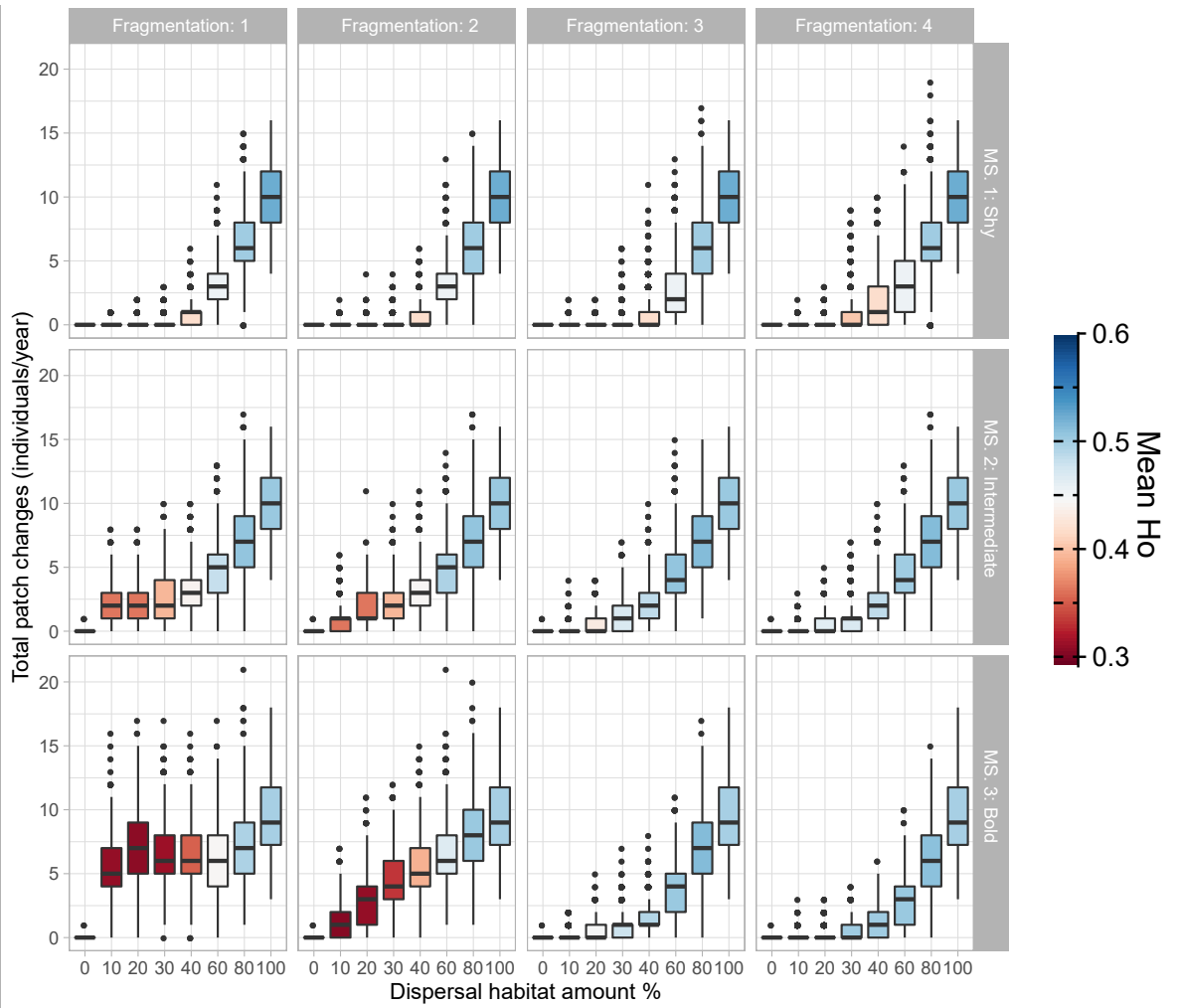

## B: Gender specific

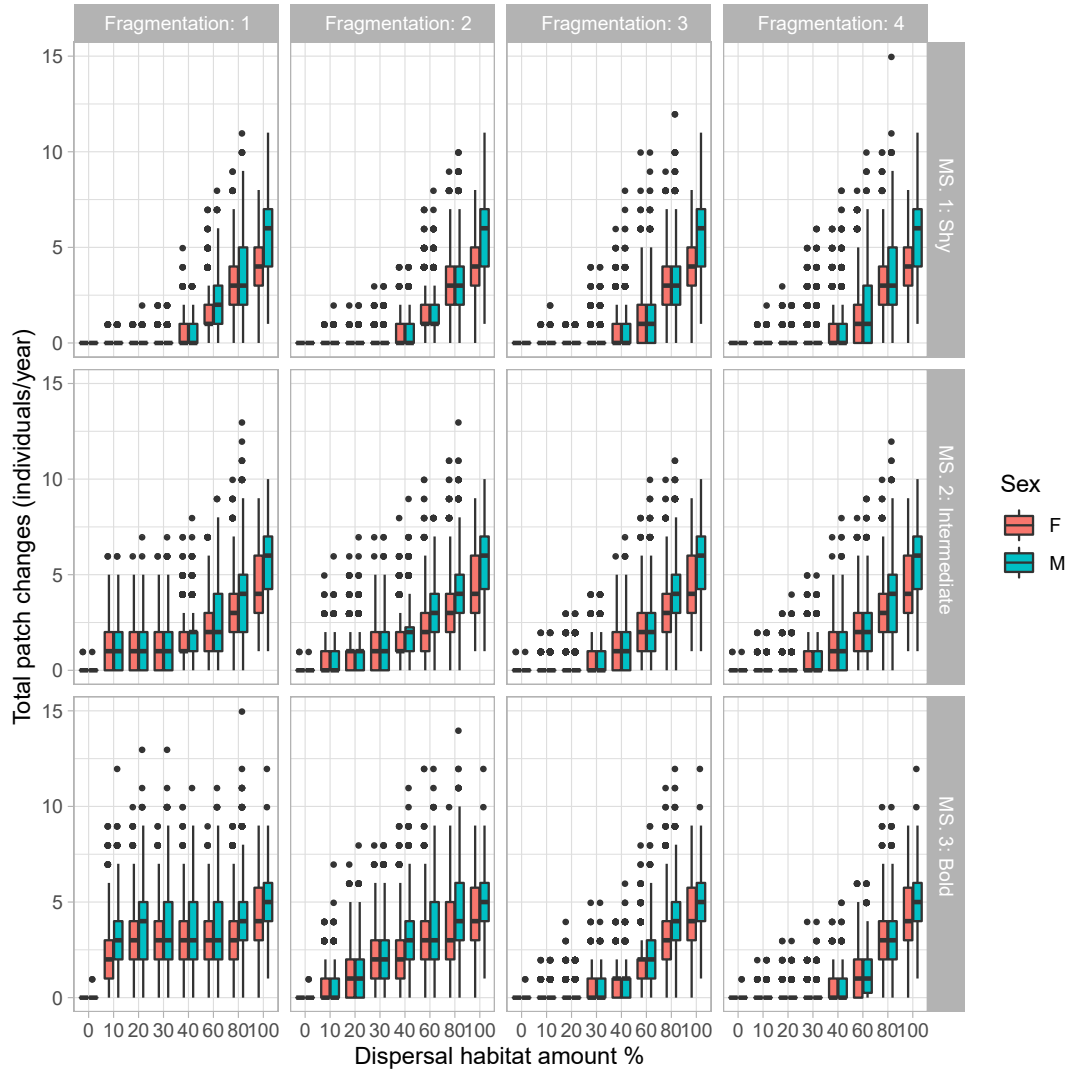

Supplement: Supplementary file 5 — Additional file 5: Figure S5. Number of disperser individuals changing breeding patch in the 200th year as a proxy for connectivity, by gender and with genetic diversity Ho. [file 40462_2020_204_MOESM5_ESM.pdf]

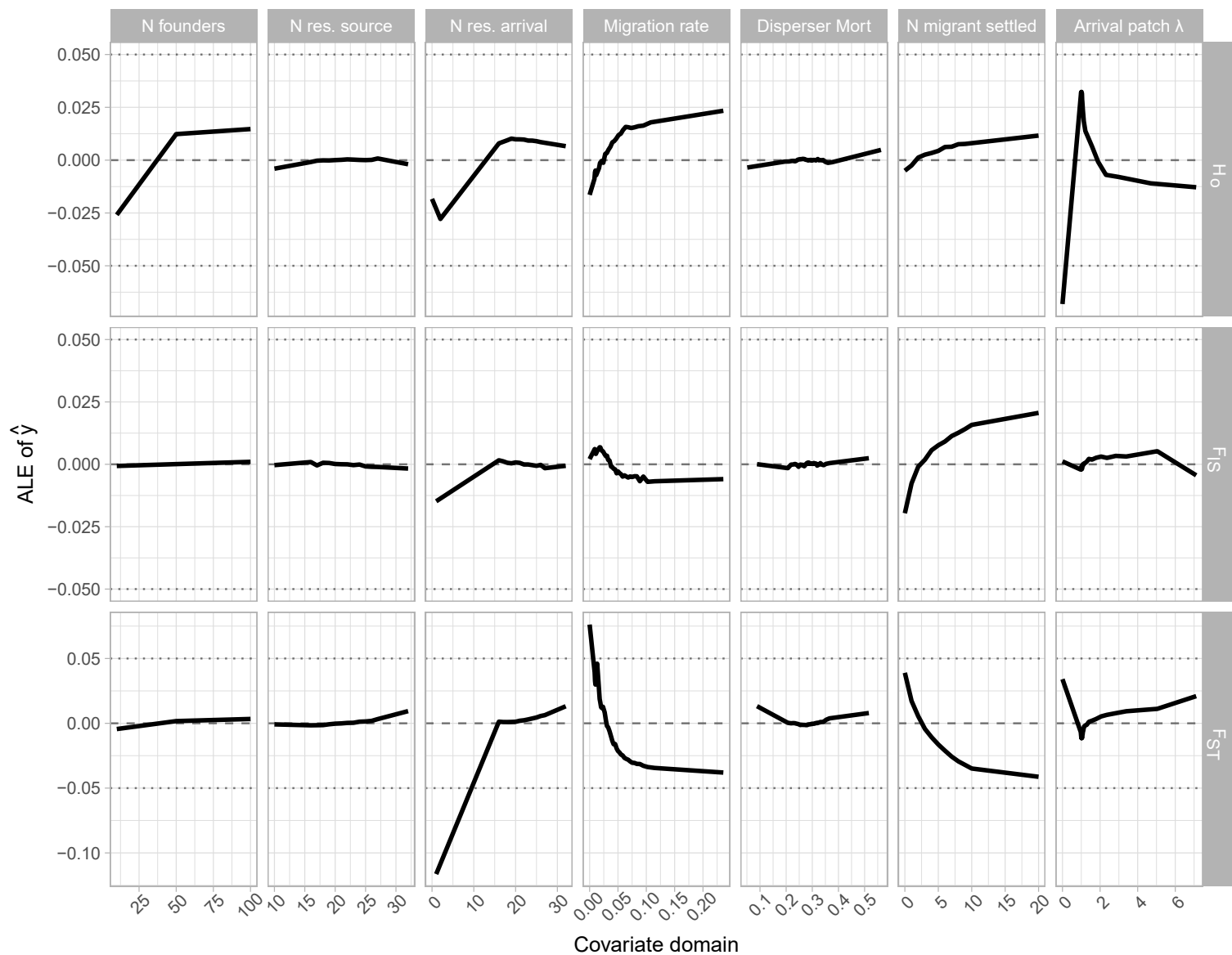

Supplement: Supplementary file 6 — Additional file 6: Figure S6. Accumulated Local Effects (ALE) plots for RF regression analysis of genetic outputs Ho, FST and FIS, using demographic covariates. [file 40462_2020_204_MOESM6_ESM.pdf]

Number of founder individuals: 10

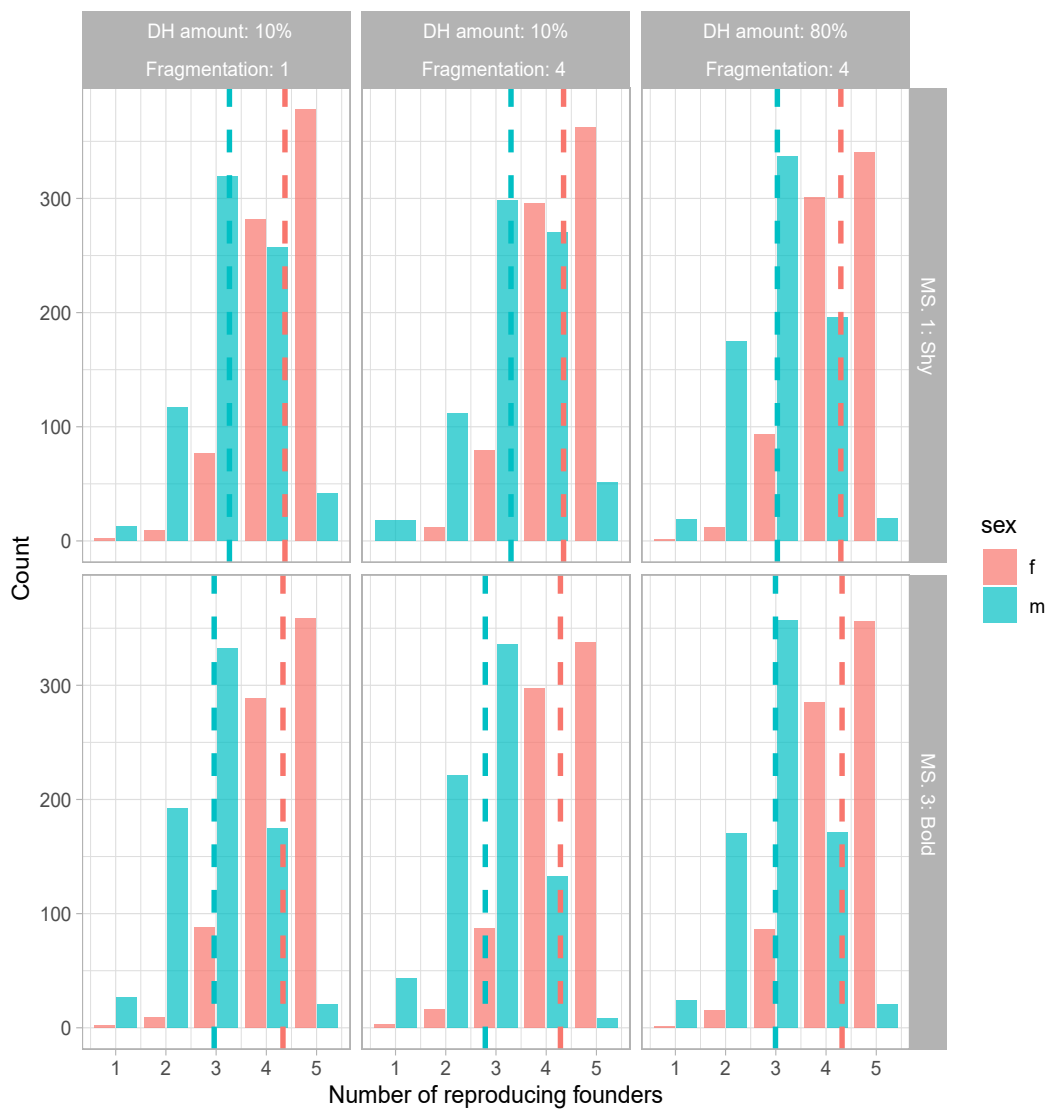

Number of founder individuals: 50

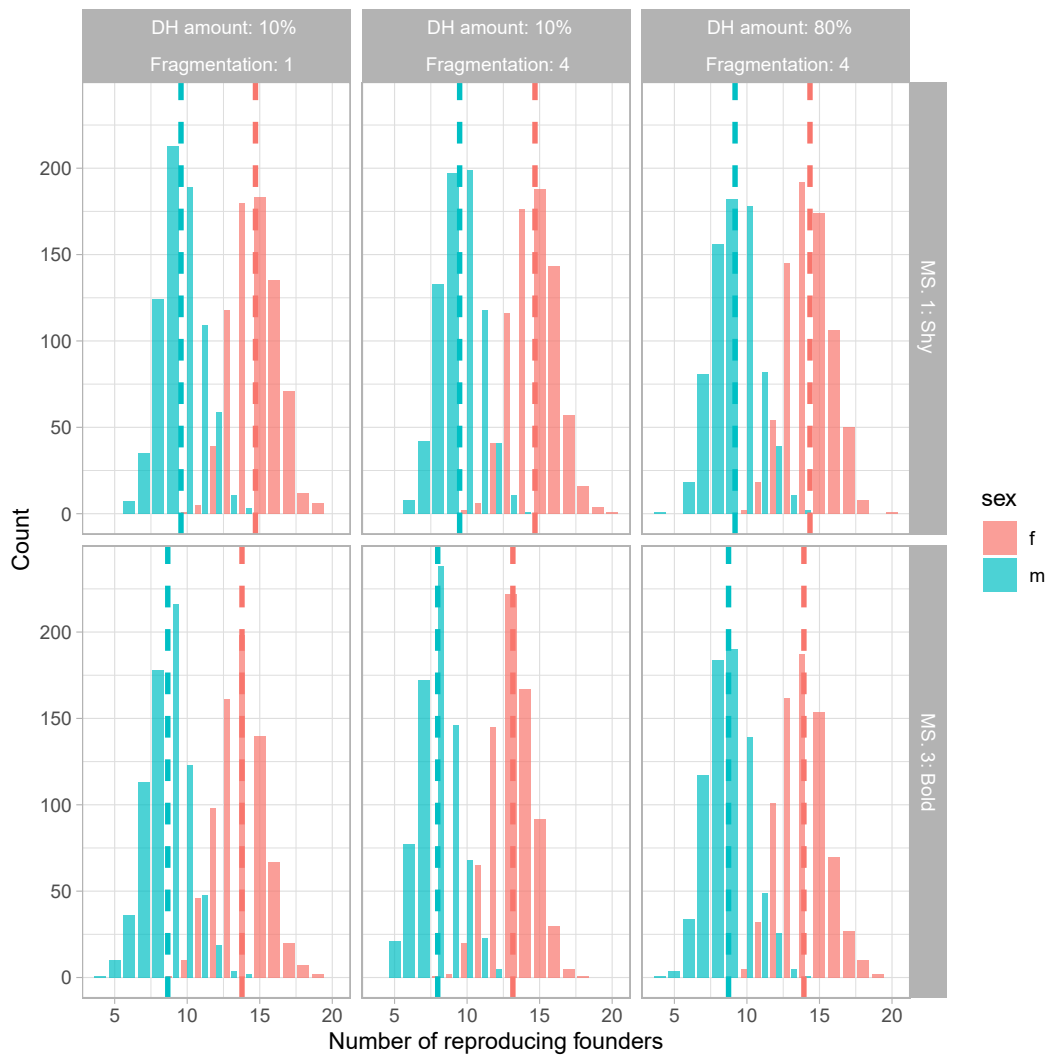

Supplement: Supplementary file 7 — Additional file 7: Figure S7. Number of founder individuals taking part in reproduction by gender, for all simulation runs of a subset of parameter space (see S2.1). For A – 10 founders, and B – 50 founders. The mean values indicated by the dotted line. [file 40462_2020_204_MOESM7_ESM.pdf]

# Number of founder individuals: 10

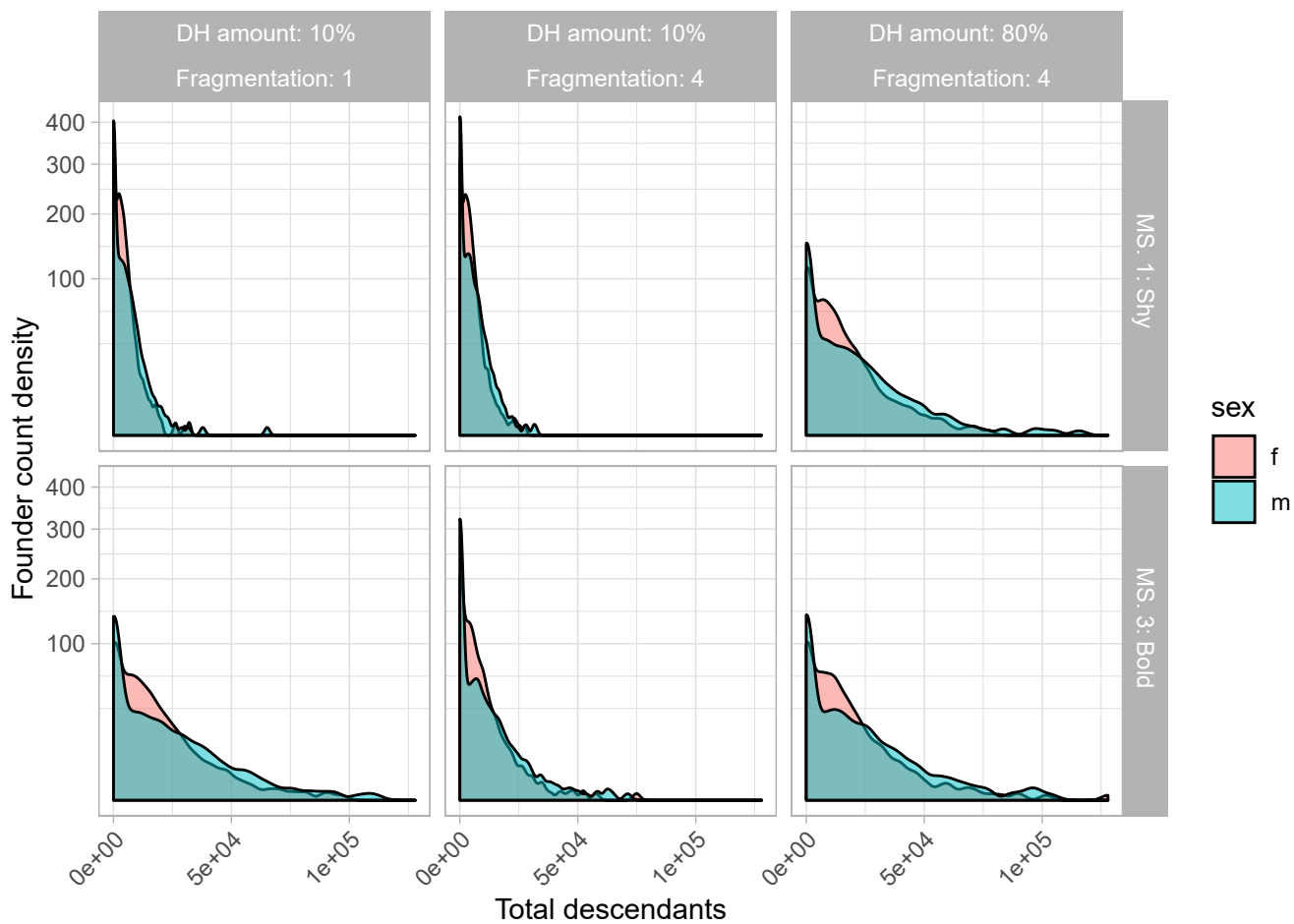

# Number of founder individuals: 50

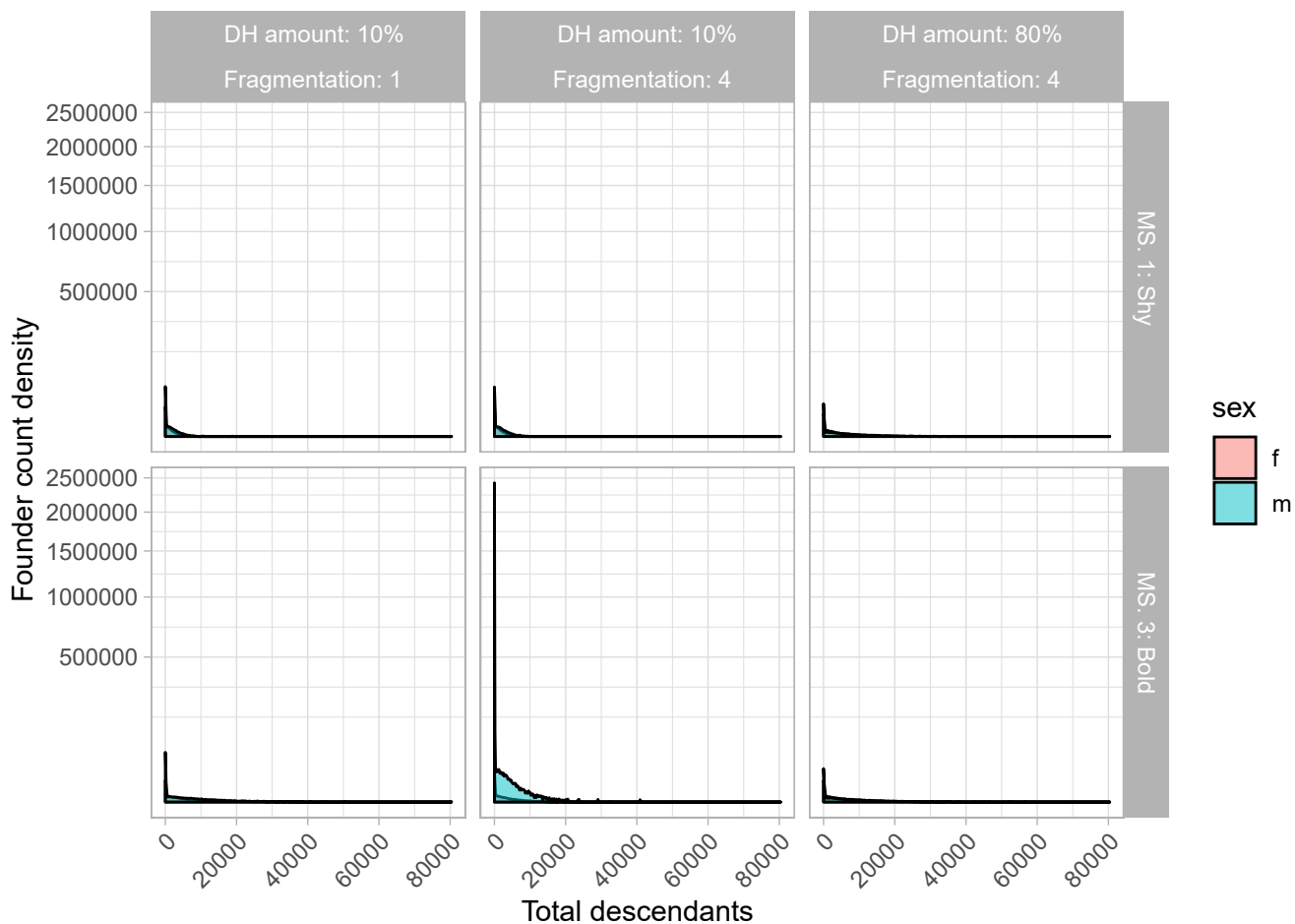

Supplement: Supplementary file 8 — Additional file 8: Figure S8. Density distribution of total number of descendants of founder individuals 50 years after reintroduction, by gender, for all simulation runs of a subset of parameter space (see S2.1). For A – 10 founders, and B – 50 founders. [file 40462_2020_204_MOESM8_ESM.pdf]

## Number of founder individuals: 10

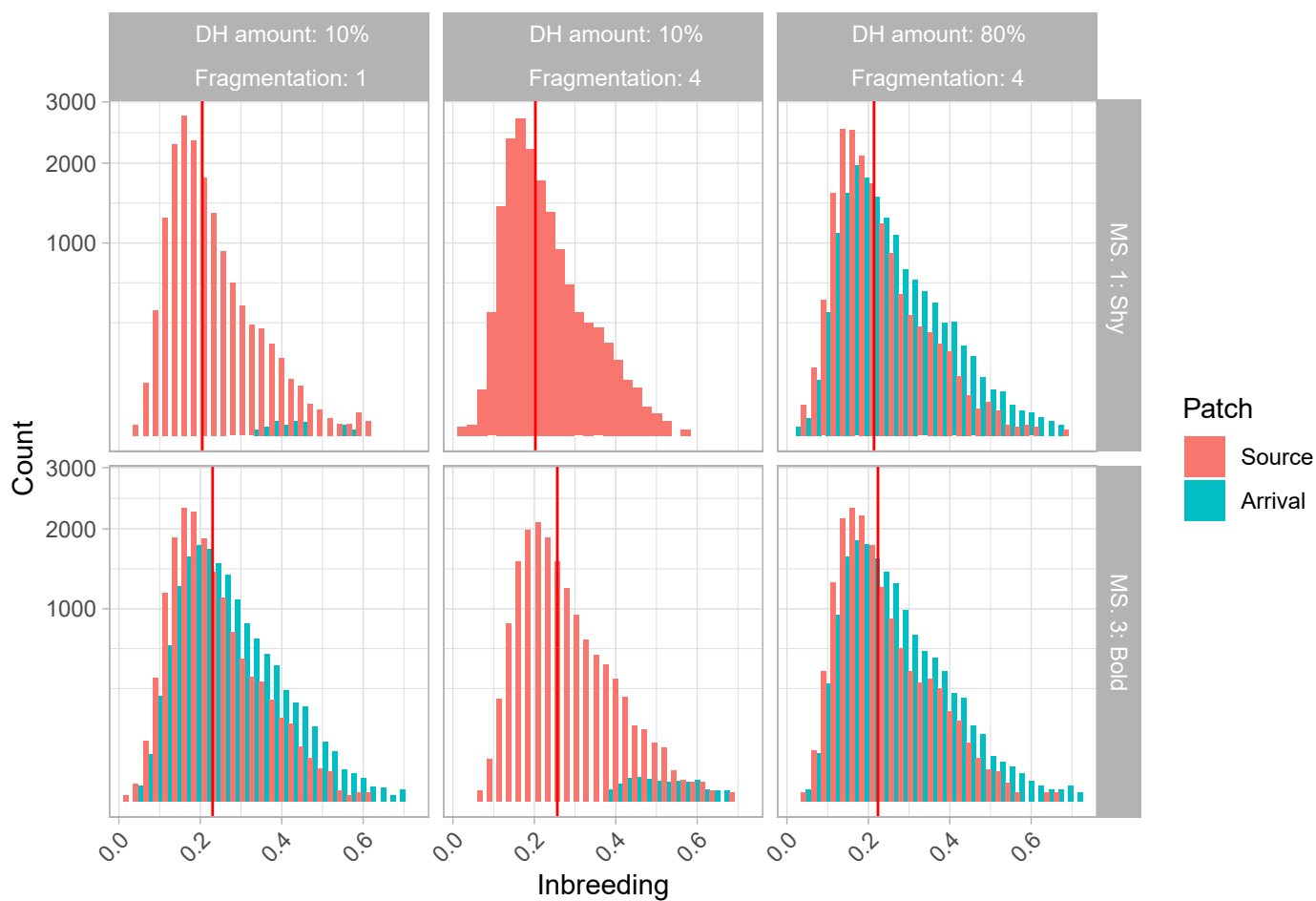

## Number of founder individuals: 50

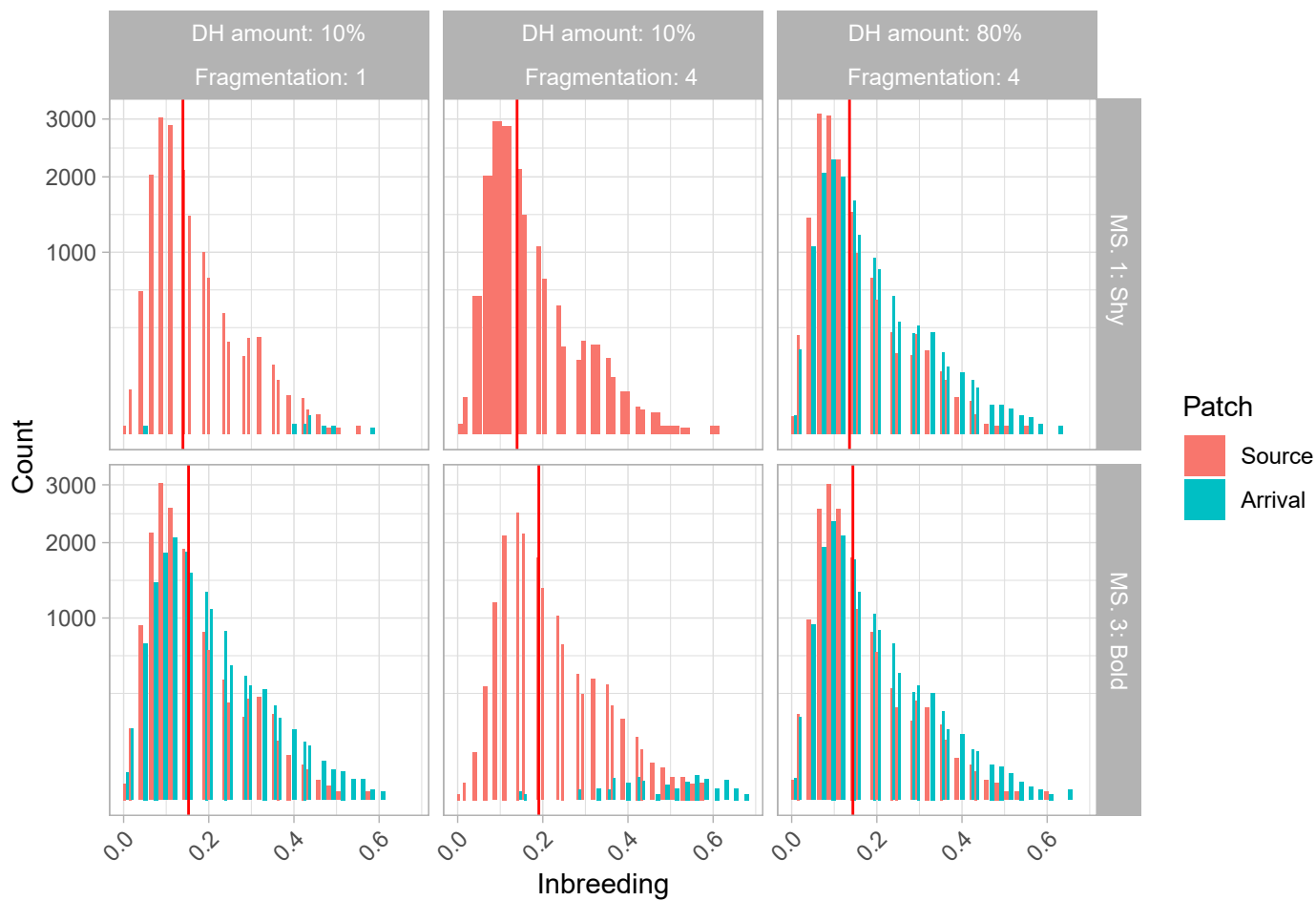

Supplement: Supplementary file 9 — Additional file 9: Figure S9. Individual inbreeding values of living individuals 50 years after reintroduction, by patch (1 = “source”, 2 = “arrival”), for all simulation runs of a subset of parameter space (see S2.1). For A – 10 founders and B – 50 founders. Inbreeding calculated by pedigree construction. Mean Inbreeding indicated by red line. [file 40462_2020_204_MOESM9_ESM.pdf]
